# Efficacy and Safety of Midazolam Oral Solution for Sedative Hypnosis and Anti-anxiety in Children: A Systematic Review and Meta-Analysis

**DOI:** 10.3389/fphar.2020.00225

**Published:** 2020-03-18

**Authors:** Xiao Cheng, Zhe Chen, Lingli Zhang, Peipei Xu, Fang Qin, Xuefeng Jiao, Yiyi Wang, Mao Lin, Linan Zeng, Liang Huang, Dan Yu

**Affiliations:** ^1^Department of Pharmacy, West China Second University Hospital, Sichuan University, Chengdu, China; ^2^Evidence-Based Pharmacy Center, West China Second University Hospital, Sichuan University, Chengdu, China; ^3^Key Laboratory of Birth Defects and Related Diseases of Women and Children, Sichuan University, Ministry of Education, Chengdu, China; ^4^West China School of Pharmacy, Sichuan University, Chengdu, China; ^5^Department of Pediatrics, West China Second University Hospital, Sichuan University, Chengdu, China

**Keywords:** midazolam oral solution, sedative hypnosis, anti-anxiety, child, systematic review, meta-analysis

## Abstract

**Background:** Midazolam is recommended by health guidelines for sedation and hypnosis in children. Oral solution is a suitable dosage form for children. But there is no conclusive evidence for sedative-hypnosis and antianxiety effects by midazolam oral solution in children.

**Methods:** Relevant studies were identified through searching PubMed, Embase, Cochrane Library, CINAHL, International Pharmaceuticals, four Chinese electronic databases, and relevant lists. Two reviewers independently selected trials, assessed trial quality, and extracted the data.

**Results:** Eighty-nine randomized controlled trials (RCTs) comparing midazolam oral solution with placebo or blank (*n* = 33), dexmedetomidine (*n* = 15), ketamine (*n* = 11), different midazolam doses (*n* = 10), midazolam injection (*n* = 8), chloral hydrate (*n* = 7), diazepam (*n* = 5), N_2_O (*n* = 5), triclofos (*n* = 4), butorphanol (*n* = 2), fentanyl (*n* = 2), hydroxyzine (*n* = 1), and thiopental (*n* = 1) were identified. Meta-analysis showed no significant difference in the success rate and duration of sedation and hypnosis between midazolam oral and injectable solution (*P* > 0.05). The success rate of sedation and hypnosis of midazolam was higher than that of ketamine [risk ratio (RR) = 1.32, 95% CI (1.07, 1.62), *I*^2^ = 0%, *P* < 0.01]. No significant difference was found in the success rate of sedation and hypnosis, mask acceptance, and parental separation between midazolam oral solution and dexmedetomidine (*P* > 0.05), and the result of one cohort study was consistent. The results of RCTs and a prospective cohort study showed that the incidence of adverse drug reactions (ADR) was 19.57% (189/966). Incidence of adverse reactions between dose groups of (0.25, 0.5] and (0.5, 1.0] mg/kg was similar [Pf (95% CI) = 0.10 (0.04, 0.24) and Pf (95% CI) = 0.09 (0.02, 0.39), respectively], higher than that of the dose group of (0, 0.25] mg/kg [Pf (95% CI) = 0.01 (0.00, 0.19)].

**Conclusions:** Available evidence suggests that midazolam oral solution is as good as midazolam injection and dexmedetomidine and is better than ketamine. Based on efficacy and safety results, an oral midazolam solution dose of 0.5–1 mg/kg is recommended for children.

## Introduction

Data from World Bank showed that children (aged 14 years and younger) accounted for 25.79% of the world's total population in 2018. The use of sedative-hypnotic drugs in assisting children to complete medical examinations and surgery has become more and more extensive (Chinese Medical Association, 2011).

Midazolam is an imidazole benzodiazepine that has an inhibitory effect on the central nervous system, which is used for examination, diagnosis, and pretreatment sedation. Midazolam is rapidly and completely absorbed after oral administration and will take effect within 10–30 min of intake. Hydroxylated by cytochrome P450 and CYP3A isoenzymes, midazolam has 1′-hydroxymidazolam as a major oxidation product (de Wildt et al., 2002). According to (WHO, 2011) and European Medicine Agency (2006) requirements for children's appropriate preparations, oral solutions are suitable for children.

Five guides (Mace et al., 2004; National Institute for Health Care Excellence, 2010, 2016; Editorial Board of Chinese Journal of Pediatrics, 2015; Chinese Medical Association Anesthesia Branch, 2017a) and three expert consensuses (Chinese Medical Association Pediatrics Branch Emergency Study Group, 2014; Chinese Medical Association Anesthesia Branch, 2017b,c) recommend midazolam for children to calm, hypnotize, and counter anxiety. The NICE guidelines recommend oral administration of midazolam for sedation before painful examination in children (National Institute for Health Care Excellence, 2010). Midazolam is included in the WHO, UK, and Indian children's formulas (World Health Organization Regional Office for South-East Asia New Delhi, 2011; Committee P F., 2017; WHO Expert Committee on the Selection Use of Essential Medicines, 2017). The UK Formulary recommends taking 0.5 mg/kg of midazolam (maximum dose 20 mg) orally in children 30–60 min before the test (Committee P F., 2017). Seven related systematic reviews were identified (Peng et al., 2014; Sun et al., 2014; Zhang et al., 2014; Guo, 2015; Pasin et al., 2015; Jun et al., 2017; Mataftsi et al., 2017). Midazolam has a lower success rate of sedative hypnosis compared to chloral hydrate, but there is no statistical difference in safety, and the quality of included studies is poor (Mataftsi et al., 2017). Dexmedetomidine is better than midazolam in children separated from parents in pre-anesthesia induction (Peng et al., 2014; Sun et al., 2014; Zhang et al., 2014; Guo, 2015; Pasin et al., 2015; Jun et al., 2017). Another study suggests that preoperative sedative and anxiolytic effects of dexmedetomidine by nasal drip are comparable to those of midazolam (Guo, 2015). Dexmedetomidine slows heartbeat, lowers blood pressure, and prolongs sedation duration (Zhang et al., 2014).

There was no systematic review of the efficacy and safety of midazolam oral solutions for sedative hypnosis and antianxiety effects in children. This study systematically evaluated the efficacy and safety of midazolam oral solution based on original research evidence and compared the effectiveness of oral and injectable solutions of midazolam. The relationship between the dose of midazolam and its effectiveness and safety was evaluated.

## Methods

### Search Strategy

PubMed, Embase, Cochrane Library, CINAHL, International Pharmaceuticals, China National Knowledge Infrastructure (CNKI), Chinese Biomedical Literature Database (CBM), Wanfang Database, VIP Database for Chinese Technical Periodicals (VIP), the WHO Clinical Trials Registry Platform, Cochrane Central Registry of Controlled Trials, and ClinicalTrials.gov were searched from their inception to August 2018. The retrieval strategy was specific and different for each database, including a combination of medical subject headings and free text terms for (“midazolam” or “dormicum” or “versed”) and (“child” or “newborn” or “infant” or “neonate” or “toddler” or “teenager” or “adolescent” or “pediatric”). We systematically searched the official website of the National Drug Administration and the Center for Adverse Reaction Monitoring for reports of midazolam adverse reactions in countries and regions around the world.

### Inclusion/Exclusion Criteria

A report was selected for inclusion if (a) participants were children aged 0–18 years; (b) the intervention group only used midazolam oral solution, and the route of administration was oral; (c) for comparisons, the control group was blank control, placebo, midazolam injection (intravenous, intramuscular, and subcutaneous), or other sedative-hypnotic drugs, and the dose and course of treatment were not limited; (d) studies focused on the efficacy and safety outcome of midazolam; (e) studies were randomized controlled trials (RCTs), cohort studies, case–control studies, case series studies, case reports, and cross-sectional survey studies.

Studies were excluded if (a) they were repeated published studies; (b) they were non-Chinese and non-English studies; (c) their full text is not available; (d) they were comparative studies with different routes of administration of midazolam (except for oral vs. injectable).

### Outcome Parameters

The primary outcome was the success rate of sedation and hypnosis (the ratio of the number of people who successfully completed an examination or surgery to the total number of people). Secondary outcomes were depth of sedation (sedative hypnosis depth scores), anxiety scores, duration of sedation and hypnosis (the time when children fell asleep to the time responding to command), the time of falling asleep (the time from the end of the medication to the state of falling into sleep), and the type and incidence of adverse reactions.

### Data Extraction

Two reviewers (Cheng and Xu) independently screened the titles and abstracts of every record. Full articles were obtained when either information conformed to satisfy the selection criteria outlined previously or not enough to ascertain because of limited information. Data were independently extracted by each reviewer and entered into a standardized form. The data extraction form included general characteristics and outcome measurements. Discrepancies were resolved by a third researcher, Chen.

### Data Analysis

Results for dichotomous outcomes were expressed as risk ratios (RR) with 95% confidence intervals (CIs), and for continuous outcomes, the mean difference (MD) with 95% CIs was accounted for. *P* ≤ 0.05 was considered statistically significant. Final outcomes of treatment vs. placebo or other medicines were used for the analysis, as recommended by the Cochrane Handbook for Systematic Review of Interventions, except where large pretreatment differences were identified; for these studies, the change from baseline was compared instead to prevent skewing of results. Where mean and/or standard deviation values were not reported, these were calculated based on reported CIs or *P*-values.

The meta-analyses were performed using Review Manager 5.3 (Copenhagen: The Nordic Cochrane Centre, The Cochrane Collaboration) software. Descriptive analysis was employed for data that cannot be meta-analyzed. The incidence of adverse reactions was analyzed by meta-analysis of uncontrolled dichotomous data. Pf[95% CI] referred to the effect volume after correcting the relevant factors. Pf was equal to odds ratio (OR) divided by (1 + OR). With reference to MedDRA 20.1, the types of adverse reactions were divided into cardiovascular system, digestive system, nervous system, and so on. In combining the studies, the conservative random effects model was employed, since the underlying effects can differ across studies and populations that are not necessarily homogeneous (DerSimonian and Laird, 1986). Statistical heterogeneity was analyzed by χ^2^ tests. Heterogeneity was quantified, where 25% = small, 50% = moderate, and 75% = high heterogeneity (Higgins, 2011). To cope with the potential heterogeneity across studies, subgroup analyses were conducted.

RCTs and cohort studies were included when analyzing the effectiveness of midazolam. Case–control studies and case reports were also included in the safety analysis.

### Assessment of Bias

The risk of bias was assessed for all clinical trials included in the quantitative and qualitative analysis. Bias of RCTs was assessed by using the Cochrane Handbook for Systematic Review of Interventions. As per recommendations in the Cochrane Handbook for Systematic Review of Interventions version 5.3, bias was assessed based on the following six domains: (1) sequence generation, (2) allocation concealment, (3) blinding, (4) incomplete outcome data, (5) selective outcome, and (6) other biases. The Newcastle–Ottawa Scale (NOS) was used to assess the quality of cohort studies and case–control studies. Critical appraisal checklists of the Joanna Briggs Institute (JBI) were used to assess the bias of case reports and case series. Two researchers (Cheng and Xu) independently completed the quality evaluation, and discrepancies were resolved by the third researcher Chen.

## Results

### Results of the Literature Search

**Figure 1:**
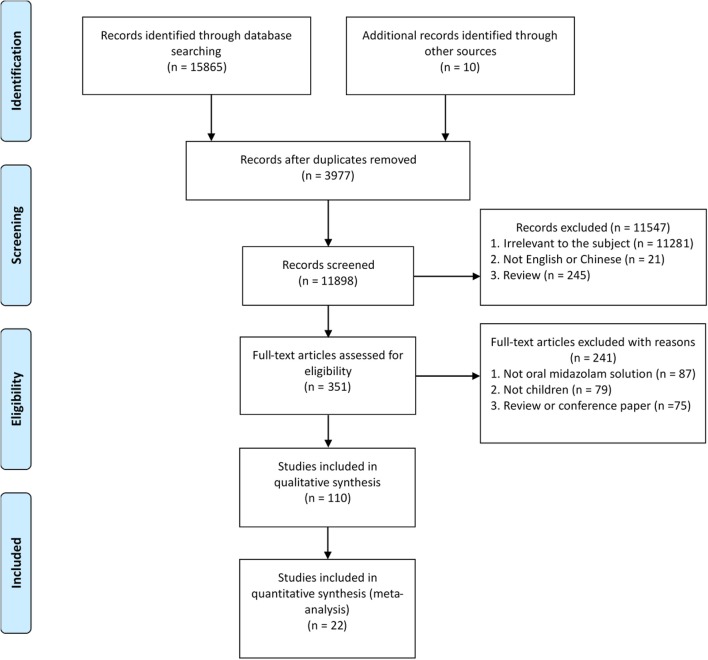
Flowchart of meta-analysis.

The search yielded a total of 15,865 references (duplication = 3,977 references). We excluded 11,547 references after reviewing the title and abstract. A further 241 references were excluded after full-text reviews, because 87 involved combination therapy or did not concern midazolam oral solution, 79 did not concern children, and 75 were review or conference papers. In total, we identified 89 RCTs, eight cohort studies, 12 case series, and one case report that met our inclusion criteria (Figure 1).

### Study Characteristics

Eighty-nine RCTs evaluating the effect and safety of midazolam oral solution with placebo or blank (*n* = 33), dexmedetomidine (*n* = 15), ketamine (*n* = 11), midazolam injection solution (*n* = 8), chloral hydrate (*n* = 7), diazepam (*n* = 5), N_2_O (*n* = 5), triclofos (*n* = 4), butorphanol (*n* = 2), hydroxyzine (*n* = 1), fentanyl (*n* = 2), thiopental (*n* = 1), and different midazolam oral solution doses (*n* = 10) were identified. Eight cohort studies evaluating the effect and safety of midazolam oral solution with placebo or blank (*n* = 4), chloral hydrate (*n* = 1), dexmedetomidine (*n* = 1), and different midazolam oral solution doses (*n* = 3) were identified. Of these 89 RCTs, the sample size ranged from 10 to 442 (median 60). The location of the first author had the following distribution: India (19/89, 21.3%), United States (15/89, 16.9%), China (10/89, 11.2%), Iran (9/89, 10.1%), UK (8/89, 9.0%), Japan (3/89, 3.4%), Canada (3/89, 3.4%), Turkey (3/89, 3.4%), Israel (3/89, 3.4%), Australia (2/89, 2.2%), Brazil (2/89, 2.2%), the United Arab Emirates (1/89, 1.1%), the Sultanate of Oman (1/89, 1.1%), Ireland (1/89, 1.1%), Germany (1/89, 1.1%), Netherlands (1/89, 1.1%), the State of Kuwait (1/89, 1.1%), Mexico (1/89, 1.1%), the Republic of South Africa (1/89, 1.1%), Nepal (1/89, 1.1%), Thailand (1/89, 1.1%), Uruguay (1/89, 1.1%), and Italy (1/89, 1.1%). Only one trial was a multicenter RCT. A total of 7,457 children were included in the 89 RCTs for effectiveness and safety analysis. Twenty-two RCTs were included in the current meta-analysis. Other characteristics of the studies were summarized in Supplementary Table 1.

### Quality Assessment

Quality assessment for RCTs, 45% (40/89) of studies used an adequate method of random sequence generation. Thirteen percent (12/89) of studies implemented adequate allocation concealment. Fifty-two percent (46/89) used the methods of blinding to patients and researchers. Thirty-nine percent (35/89) used the methods of blinding to the outcome measurer. The mean score of risk of bias of eight cohort studies was 5.75. Results of quality assessment of 12 case series and one case report were in Supplementary Tables 2–5.

## Effectiveness Outcomes

### Success Rate of Sedation and Hypnosis

Four RCTs (Payne et al., 1991; McCluskey and Meakin, 1994; Liacouras et al., 1998; Azarfar et al., 2014) and one cohort study (Keles and Kocaturk, 2018) compared midazolam oral solution with blank or placebo, including 325 and 1,504 children, respectively. Oral solution of midazolam was better, for RCTs, RR=1.61, 95% CI (1.16, 2.24), *I*^2^ = 78%, *P* < 0.01, and for cohort studies, RR = 1.41, 95% CI (1.24, 1.60), *P* < 0.01.

Two RCTs (Payne et al., 1991; Khodadad et al., 2016) made a comparison between midazolam oral and injectable solutions, with 186 children included. RCTs showed no significant difference [RR = 1.01, 95% CI (0.93, 1.10), *I*^2^ = 0%, *P* > 0.05].

One RCT (Pisalchaiyong et al., 2005), with 26 children included, compared midazolam oral solution with diazepam, showing a higher success rate than diazepam [RR = 1.59, 95% CI (1.03, 2.45), *P* < 0.05].

Midazolam oral solution and chloral hydrate were compared. For the invasive procedure, one RCT (Derakhshanfar et al., 2013) with 160 children demonstrated that the success rate of sedation and hypnosis of midazolam oral solution was lower [RR = 0.78, 95% CI (0.68, 0.91), *P* < 0.01]. For the non-invasive procedure, three RCTs (D'agostino and Terndrup, 2000; Wheeler et al., 2001; Hijazi et al., 2014) with 359 children proved that the success rate of midazolam oral solution was lower [RR = 0.39, 95% CI (0.28, 0.54), *I*^2^ = 51%, *P* < 0.01]. One cohort study (Schmalfuss, 2005) with 326 children certified that midazolam oral solution had a lower success rate [RR = 0.60, 95% CI (0.39, 0.93), *P* < 0.05].

One RCT (Radhika et al., 2016), with 60 children included, was about comparing midazolam oral solution with triclofos. The RCT showed that the oral solution of midazolam was more successful [RR = 1.56, 95% CI (1.14, 2.12), *P* < 0.01].

Two RCTs (Chen, 2009; Rubinstein et al., 2016) with 88 children included compared midazolam oral solution with ketamine. The RCTs indicated that the oral solution of midazolam was more successful [RR = 1.30, 95% CI (1.06, 1.59), *I*^2^ = 0%, *P* < 0.05].

Four RCTs (Talon et al., 2009; Arora et al., 2014; Ghai et al., 2017; Kumari et al., 2017) and one cohort study (Keles and Kocaturk, 2018) compared midazolam oral solution with dexmedetomidine, including 275 and 52 children, respectively. RCTs showed that the difference was not statistically significant [RR = 0.91, 95% CI (0.71, 1.15), *I*^2^ = 88%, *P* > 0.05]. The cohort study also showed no statistical significance [RR = 0.96, 95% CI (0.87, 1.07), *P* > 0.05].

**Figure 2:**
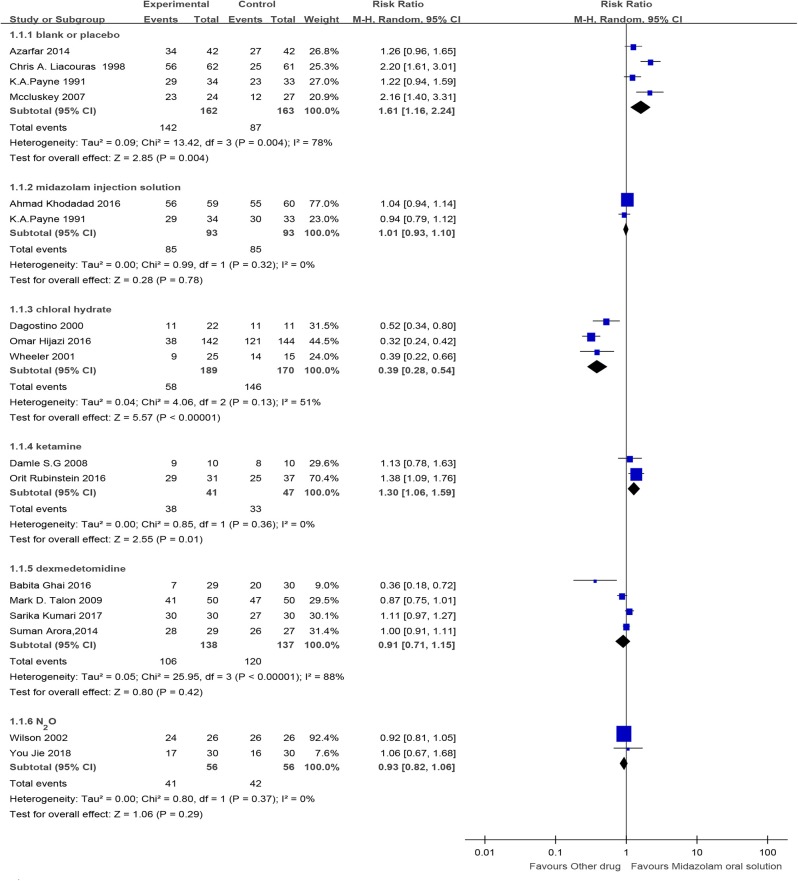
Forest plot for the success rate of sedation and hypnosis of midazolam oral solution.

Two RCTs (McErlean et al., 2003; You et al., 2018) with 86 children included compared midazolam oral solution with N_2_O. RCTs proved that the difference was not statistically significant [RR = 0.93, 95% CI (0.82, 1.06), *I*^2^ = 0%, *P* > 0.05] (Figure 2).

A total of 10 case series (Soy et al., 1994; Kil et al., 2003; Day et al., 2006; Jing et al., 2009, 2010; Lourenço-Matharu and Roberts, 2010; Xia et al., 2010; Sun et al., 2011; Ma et al., 2012; Dighe, 2014) reported success rate of sedation and hypnosis, with 931 children included. The success rate of sedation and hypnosis of midazolam oral solution was 744/969 (76.78%).

### Depth of Sedation

Five RCTs (McMillan et al., 1992; Mitchell et al., 1997; Mishra et al., 2005; Wan et al., 2005; Azevedo et al., 2013) and one cohort study (Aykut and Işik, 2018) compared midazolam oral solution with blank or placebo, including 365 and 64 children, respectively. Three RCTs showed the depth of sedation of midazolam oral solution was deeper [MD = 0.56–2.34, *P* < 0.01]. The remaining two RCTs showed no significant difference [MD = 0.04–0.40, *P* > 0.05]. The cohort study showed the depth of sedation of midazolam oral solution was deeper [MD = 1.42, 95% CI (1.01, 1.83), *P* < 0.01].

Two RCTs (Phadke et al., 2014; Khodadad et al., 2016) compared midazolam oral with injectable solution, including 391 children. One RCT showed no significant difference in the sedation score [MD = 0.20, 95% CI (−0.04, 0.44), *P* > 0.05]. Another showed that the depth of sedation of midazolam oral solution was deeper [MD = 0.20, 95% CI (0.02, 0.38), *P* < 0.05].

Two RCTs (Haas et al., 1996; Derakhshanfar et al., 2013) compared midazolam oral solution with chloral hydrate, including 206 children. One RCT showed that the depth of sedation of midazolam oral solution was deeper [MD = 0.43, 95% CI (0.33, 0.53), *P* < 0.01]. Another indicated that the depth of sedation of chloral hydrate was deeper [MD = −0.91, 95% CI (−1.17, −0.65), *P* < 0.01].

One RCT (Singh et al., 2003), including 60 children, showed no significant difference in the sedation score between midazolam oral solution and triclofos [MD = −0.23, 95% CI (−0.55, 0.09), *P* > 0.05].

Three RCTs (Debnath and Pande, 2003; Sen et al., 2013; Rubinstein et al., 2016) compared midazolam oral solution with ketamine, with 166 children included. One showed the depth of sedation of midazolam oral solution was deeper [MD = −0.90, 95% CI (−1.36, −0.44), *P* < 0.01]. Two RCTs proved no significant difference [MD = −0.31 to 0.10, *P* > 0.05].

Two RCTs (Singh et al., 2005; Sinha et al., 2012) compared midazolam oral solution with butorphanol, including 120 children. RCTs manifested that the depth of sedation of butorphanol was deeper [MD = −0.84 to 0.43, *P* < 0.05].

One RCT (Hua et al., 2012), including 20 children, showed that the depth of sedation of midazolam oral solution was deeper than that of fentanyl [MD = 0.60, 95% CI (0.04, 1.16), *P* < 0.05].

Four RCTs (Ghali et al., 2011; Liu et al., 2014; Faritus et al., 2015; Kumari et al., 2017) compared midazolam oral solution with dexmedetomidine, including 300 children. Two RCTs demonstrated no significant difference [MD = −0.07 to 0.08, *P* > 0.05]. One RCT showed that the depth of sedation of dexmedetomidine was deeper [MD = 1.05, 95% CI (0.52, 1.58), *P* < 0.01]. Another showed that the depth of sedation of midazolam oral solution was deeper [MD = 0.75, 95% CI (0.54, 0.96), *P* < 0.01].

### Duration of Sedative Hypnosis

Two RCTs (Payne et al., 1991; Khodadad et al., 2016) compared midazolam oral solution with injection, including 180 children, and showed no significant difference [MD = −0.26, 95% CI (−2.76, 2.23), *I*^2^ = 0%, *P* > 0.05].

Midazolam oral solution and chloral hydrate were compared. For the invasive procedure, one RCT (Derakhshanfar et al., 2013), with 160 children included, proved that the duration of sedative hypnosis of midazolam oral solution was longer than that of chloral hydrate [MD = 23.10, 95% CI (17.39, 28.81), *P* < 0.01]. For the non-invasive procedure, four RCTs (D'agostino and Terndrup, 2000; Wheeler et al., 2001; Hijazi et al., 2014; Salehi et al., 2017), including 427 children, showed that the duration of sedative hypnosis of chloral hydrate was longer [MD = −25.79, 95% CI (−38.83, −12.74), *I*^2^ = 88%, *P* < 0.01].

**Figure 3:**
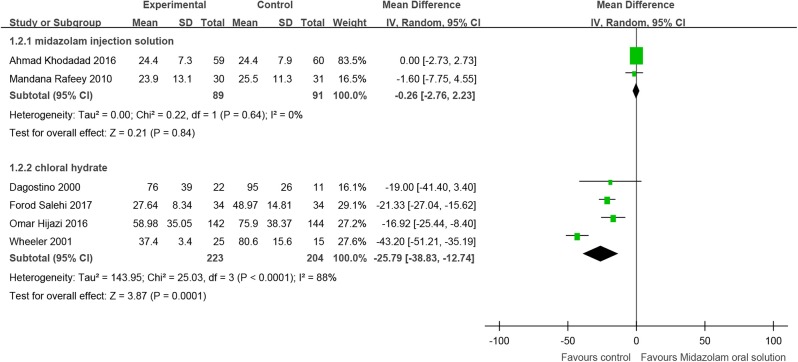
Forest plot for the duration of sedative hypnosis for midazolam oral solution.

One RCT (Singh et al., 2003) compared midazolam oral solution with triclofos, including 60 children. The RCT indicated that the duration of sedative hypnosis of midazolam oral solution was shorter [MD = −38.23, 95% CI (−44.94, −31.52), *P* < 0.01] (Figure 3).

### Time to Fall Asleep

Midazolam oral solution and chloral hydrate were compared. For the invasive procedure, one RCT (Derakhshanfar et al., 2013), with 160 children included, demonstrated that it took longer for children taking midazolam oral solution to fall asleep [MD = 14.40, 95% CI (12.09, 16.71), *P* < 0.01]. For the non-invasive procedure, three RCTs (Wheeler et al., 2001; Hijazi et al., 2014; Salehi et al., 2017) with 394 children included showed different results. One of the RCTs (Wheeler et al., 2001) showed no significant difference [MD = 2.30, 95% CI (−0.34, 4.94), *P* > 0.05]. Another RCT (Hijazi et al., 2014) showed that it took longer for children taking midazolam oral solution to fall asleep [MD = 28.82, 95% CI (21.54, 36.10), *P* < 0.01]. The last RCT (Salehi et al., 2017) showed that the time to fall asleep of midazolam oral solution was shorter [MD = −12.79, 95% CI (−15.11, −10.47), *P* < 0.01].

One RCT (Singh et al., 2003) compared midazolam oral solution with triclofos, including 60 children. The RCT indicated that the time to fall asleep of midazolam oral solution was shorter [MD = −16.10, 95% CI (−18.11, −14.09), *P* < 0.01].

**Figure 4:**
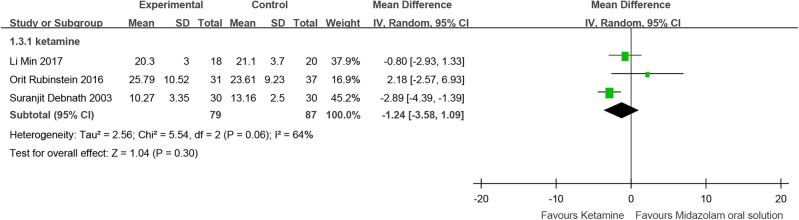
Forest plot for the time to fall asleep for midazolam oral solution.

Three RCTs (Debnath and Pande, 2003; Rubinstein et al., 2016; Li et al., 2017) compared midazolam oral solution with ketamine, including 166 children. These RCTs showed no significant difference [MD = −1.24, 95% CI (−3.58, 1.09), *I*^2^ = 64%, *P* > 0.05] (Figure 4).

One RCT (Singh et al., 2005) compared midazolam oral solution with butorphanol, including 60 children. The RCT showed that it took longer for children taking midazolam oral solution to fall asleep [MD = 5.00, 95% CI (3.78, 6.22), *P* < 0.01].

One RCT (Li et al., 2017) compared midazolam oral solution with dexmedetomidine, including 40 children, and showed no significant difference [MD = −2.20, 95% CI (−4.46, 0.06), *P* > 0.05].

### Anxiety Scores

Four RCTs compared midazolam oral solution with blank or placebo, including 244 children. Three trials (Alderson and Lerman, 1994; Silver et al., 1994; Ghai et al., 2017) showed no significant difference [MD = −0.29 to 0.60, *P* > 0.05]. One (McMillan et al., 1992) showed that the anxiety alleviation of midazolam oral solution was better [MD = 0.60 to 0.70, *P* < 0.01].

One RCT (Lyons et al., 1995) compared midazolam oral solution with thiopental, including 51 children, and showed that the anxiety alleviation of midazolam oral solution was worse [MD = −1.26, 95% CI (−1.61, −0.91), *P* < 0.01].

One RCT (Debnath and Pande, 2003) compared midazolam oral solution with ketamine, including 60 children, which showed no significant difference [MD = 0.27, 95% CI (−0.12, −0.66), *P* > 0.05].

One RCT (Ghali et al., 2011) compared midazolam oral solution with dexmedetomidine, including 120 children, which showed that the anxiety scores of midazolam oral solution were higher and that the anxiolytic effect of midazolam oral solution was worse [MD = 12.76, 95% CI (11.25, 14.27), *P* < 0.01].

One RCT (Keidan et al., 2005) compared midazolam oral solution with N_2_O, including 47 children. The RCT showed no significant difference [MD = 0.00, 95% CI (−0.86, 0.86), *P* > 0.05].

### Success Rate of Parental Separation

Two RCTs (Mishra et al., 2005; El Batawi, 2015) compared midazolam oral solution with blank or placebo, including 178 children, which indicated that the success rate of parental separation of midazolam oral solution was higher [RR = 5.67, 95% CI (3.52, 9.15), *I*^2^ = 0%, *P* < 0.01].

One RCT (Khodadad et al., 2016) compared midazolam oral solution with its injection, including 119 children, which indicated that success rate of parental separation of midazolam oral solution was higher [RR = 1.11, 95% CI (1.00, 1.24), *P* = 0.05].

One RCT (Radhika et al., 2016) compared midazolam oral solution with triclofos, including 60 children, and showed no significant difference [RR = 1.0, 95% CI (0.91, 1.10), *P* > 0.05].

**Figure 5:**
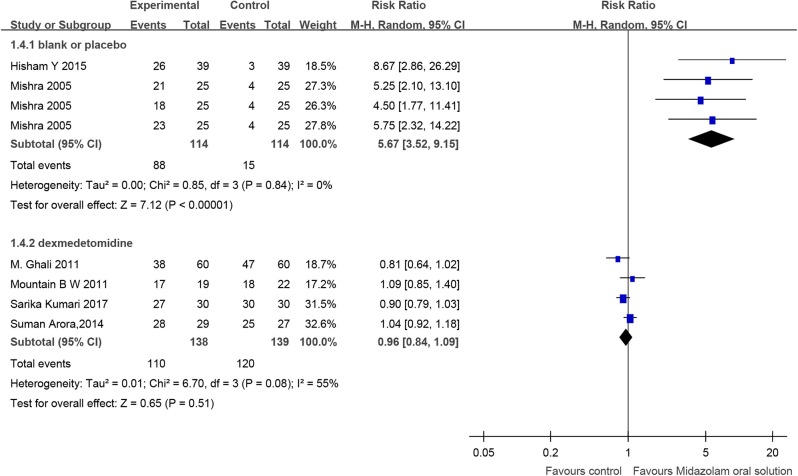
Forest plot for the success rate of parental separation of midazolam oral solution.

Four RCTs (Talon et al., 2009; Arora et al., 2014; Ghai et al., 2017; Kumari et al., 2017) and one cohort study (Keles and Kocaturk, 2018) compared midazolam oral solution with dexmedetomidine, including 277 and 52 children, respectively. The RCTs showed no significant difference [RR = 0.96, 95% CI (0.84, 1.09), *I*^2^ = 55%, *P* > 0.05]. The cohort study also showed no significant difference [RR = 1.00, 95% CI (0.85, 1.17), *P* > 0.05] (Figure 5).

### Success Rate of Mask Acceptance

One RCT (Chaudhary et al., 2014) compared midazolam oral solution with hydroxyzine, including 40 children. This RCT showed that the success rate of mask acceptance of midazolam oral solution was higher [RR = 4.56, 95% CI (2.00, 10.36), *P* < 0.01].

Two RCTs (Chaudhary et al., 2014; Radhika et al., 2016) compared midazolam oral solution with triclofos, including 40 children. One RCT showed that the success rate of mask acceptance of midazolam oral solution was higher [RR = 13.67, 95% CI (2.92, 63.98), *P* < 0.01]. Another RCT showed no significant difference [RR = 0.89, 95% CI (0.72, 1.10), *P* > 0.05].

**Figure 6:**
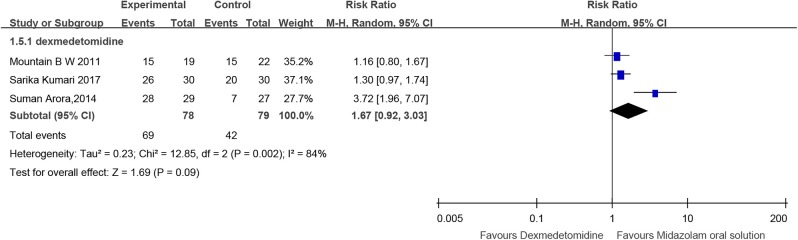
Forest plot for the success rate of mask acceptance of midazolam oral solution.

Three RCTs (Mountain et al., 2011; Arora et al., 2014; Kumari et al., 2017) and one cohort study (Keles and Kocaturk, 2018)compared midazolam oral solution with dexmedetomidine, including 157 and 52 children, respectively. RCTs showed no significant difference [RR = 1.67, 95% CI (0.92, 3.03), *I*^2^ = 84%, *P* > 0.05]. Cohort study also showed no significant difference [RR = 1.00, 95% CI (0.85, 1.17), *P* > 0.05] (Figure 6).

### Comparison of Different Doses of Oral Midazolam

According to the British National Formulary for Children and the instructions of theUS midazolam syrup(Product name:Versed), the dose of midazolam oral solution was divided into three dose groups (0–0.25), mg/kg (0.25–0.5), mg/kg and (0.5–1.0) mg/kg.

### Depth of Sedation

Three RCTs (McMillan et al., 1992; Mishra et al., 2005; Somri et al., 2012) and one cohort study (Peretz et al., 2014) compared (0.25–0.5) mg/kg and (0.5–1.0) mg/kg dose groups, including 225 and 46 children, respectively. Two RCTs showed no significant difference in the depth of sedation between two dose groups [MD = −0.18–0.08, *P* > 0.05]. One RCT showed that the depth of sedation of (0.25–0.5) mg/kg midazolam oral solution was lighter [MD = −1.45, 95% CI (−1.80, −1.10), *P* < 0.01], and the cohort study proved the same outcome [MD = −2.04, 95% CI (−2.26, −1.82), *P* < 0.01].

### Time to Fall Asleep

One RCT (Somri et al., 2012) and one cohort study (Aykut and Işik, 2018) compared (0.25–0.5] mg/kg and (0.5–1.0) mg/kg dose groups, including 90 and 46 children, respectively. The RCT showed that it took longer for children taking (0.25–0.5) mg/kg midazolam oral solution to fall asleep [MD = 5.45, 95% CI (3.76, 7.14), *P* < 0.01]. The cohort study got the same result [MD = 4.13, 95% CI (2.11, 6.15), *P* < 0.01].

### Anxiety Scores

One RCT (Chen, 2009) compared (0–0.25) mg/kg and (0.25–0.5) mg/kg dose groups, including 60 children, which showed no significant difference between two dose groups [MD = 2.50, 95% CI (−2.61, 7.61), *P* > 0.05].

One RCT (McMillan et al., 1992) with 60 children included compared (0.25–0.5) mg/kg and (0.5–1.0) mg/kg dose groups, which demonstrated no significant difference [MD = −0.08, 95% CI (−0.32, 0.16), *P* > 0.05].

### Success Rate of Parental Separation

One RCT (Mishra et al., 2005) with 75 children included compared (0.25–0.5) mg/kg and (0.5–1.0) mg/kg dose groups, which demonstrated no significant difference [RR = 0.35, 95% CI (0.10, 1.19), *P* > 0.05].

### Safety Outcomes

A total 33 studies were included, including 20 RCTs, three cohort studies, nine case series, and one case report. The results of RCTs (Weldon et al., 1992; Mitchell et al., 1997; D'agostino and Terndrup, 2000; Luhmann et al., 2001; Younge and Kendall, 2001; Debnath and Pande, 2003; Horiuchi et al., 2005; Keidan et al., 2005; Mishra et al., 2005; Yildirim et al., 2006; Ashrafi et al., 2013; Derakhshanfar et al., 2013; Hijazi et al., 2014; Liu et al., 2014; Zhai et al., 2014; Faritus et al., 2015; Khodadad et al., 2016; Li et al., 2017; Salehi et al., 2017; You et al., 2018) and prospective cohort studies (Nathan and Vargas, 2002; Schmalfuss, 2005; Peretz et al., 2014) showed 189 cases of adverse drug reaction (ADR) and that the incidence of ADR was 19.57% (189/966). ADR involved the following: (1) mental system (lethargy, restless sleep, prolonged sedation, euphoria or restlessness, irritability, agitation, abnormal behavior, mood swings, headache, aggressiveness, and inner conflicts); (2) digestive system (nausea, vomiting, and hiccups); (3) respiratory system (laryngospasm and the need of auxiliary breathing); and (4) others such as urinary incontinence, chills, nystagmus, and limb shaking. The systemic evaluation of the case series (Soy et al., 1994; Fraone et al., 1999; Kil et al., 2003; Day et al., 2006; Kain et al., 2007; Jing et al., 2009, 2010; Lourenço-Matharu and Roberts, 2010; Xia et al., 2010; Sun et al., 2011; Ma et al., 2012; Dighe, 2014) showed 106 cases of mental system adverse reactions, including 74 cases of crying agitation, nine agitation cases, six lip bite cases, six lethargy cases, five hallucinations cases, four abnormal excitement cases, one screaming case, and one case of being awakened; 29 cases of digestive system adverse reactions, including 28 snoring cases and one vomiting case; one case of respiratory system adverse reactions, snoring; 26 cases of other adverse reactions, including 18 cases of diplopia, five urinary incontinence cases, one heart rate speeding up case, one limb shaking case, and one incapable of standing case. One case report study reported (Bernardini et al., 2017) adverse reactions. After administration of 0.5 mg/kg of midazolam solution, dyspnea accompanied by wheezing, prolonged expiration time, respiratory distress and exhaustion, peripheral blood saturation <90%, and the phenomenon of nasal expansion occurred. After receipt of supplemental oxygen (5 L/min) through the mask, oral corticosteroids (betamethasone 4 mg), aerosolized short-acting beta agonist (salbutamol spray), and intravenous infusion, the symptoms resolved within 1 h. The incidence of adverse reactions of midazolam oral solution was summarized in Table 1.

**Table 1:** **Incidence of adverse reactions to midazolam oral solution**.

**ADR type**	**No. of studies included**	**No. of ADR cases**	**No. of patients**	**ADR incidence**	**Pf [95% CI]**	**Heterogeneity**	
						***I*^**2**^ (%)**	***P***
**MENTAL SYSTEM**							
Prolonged sedation	2	60	96	0.62	0.28 [0.00, 0.97]	94	<0.01
Dysphoria	3	34	157	0.22	0.25 [0.04, 0.73]	94	0.30
Agitation	3	20	128	0.16	0.16 [0.10, 0.23]	0	0.85
Abnormal behavior	2	16	125	0.13	0.15 [0.01, 0.75]	95	<0.01
Euphoria	2	11	147	0.07	0.07 [0.01, 0.42]	88	<0.01
Lethargy or disturbed sleep	2	5	58	0.09	0.09 [0.04, 0.19]	0	0.7
Irritability	2	8	128	0.06	0.07 [0.01, 0.50]	89	<0.01
Inner conflict	2	8	158	0.05	0.05 [0.03, 0.10]	0	0.65
Aggressivity	1	2	97	0.02	0.03 [0.00, 0.20]	–	–
Mood swing	1	1	97	0.01	0.01 [0.00, 0.07]	–	–
Headache	1	1	80	0.01	0.02 [0.01, 0.08]	–	–
**DIGESTIVE SYSTEM**							
Hiccup	3	6	90	0.07	0.07 [0.03, 0.15]	0	0.61
Nausea and vomiting	13	9	547	0.02	0.03 [0.02, 0.05]	0	0.87
**RESPIRATORY SYSTEM**							
Laryngospasm	1	1	30	0.03	0.03 [0.00, 0.20]	–	–
Need assisted breathing	1	1	142	0.01	0.01 [0.00, 0.05]	–	–
Other	7	6	324	0.02	0.03 [0.01, 0.10]	56	0.04

The total incidences of adverse reactions in the midazolam oral solution of (0, 0.25), (0.25, 0.5), and (0.5, 1.0) mg/kg were Pf (95% CI) = 0.01 (0.00, 0.19), 0.10 (0.04, 0.24), and 0.09 (0.02, 0.39), respectively.

Five RCTs (Weldon et al., 1992; Mitchell et al., 1997; Luhmann et al., 2001; Mishra et al., 2005; Yildirim et al., 2006) and one cohort study (Elder and Longenecker, 1995) compared midazolam oral solution with blank or placebo, including 372 and 122 children, respectively, which proved no statistical significance [RR = 0.77, 95% CI (0.21, 2.81), *I*^2^ = 29%, *P* = 0.69; RR = 6.63, 95% CI (0.41, 108.36), *P* = 0.18]. Two RCTs (Schmidt et al., 2007; Khodadad et al., 2016) comparing midazolam oral solution with injectable solution, with 179 children included, proved no statistical significance [RR = 5.00, 95% CI (0.25, 99.95), *P* = 0.29]. Five RCTs (D'agostino and Terndrup, 2000; Ashrafi et al., 2013; Derakhshanfar et al., 2013; Hijazi et al., 2014; Salehi et al., 2017) and one cohort study (Schmalfuss, 2005) compared midazolam oral solution with chloral hydrate, with 745 and 326 children included, respectively, and both proved no statistical significance [RR = 1.00, 95% CI (0.29, 3.45), *I*^2^ = 76%, *P* = 1.00; RR = 0.80, 95% CI (0.05, 12.94), *P* = 0.87]. Four RCTs (Younge and Kendall, 2001; Debnath and Pande, 2003; Horiuchi et al., 2005; Li et al., 2017) comparing midazolam oral solution with ketamine, with 210 children included, proved that the incidence of adverse reactions in midazolam oral solutions was higher [RR = 1.71, 95% CI (1.24, 2.35), *I*^2^ = 0%, *P* = 0.001]. For the incidence of adverse effects of the mental system, midazolam oral solution was higher [RR = 2.84, 95% CI (1.11, 7.28), *I*^2^ = 58%, *P* = 0.03] (Younge and Kendall, 2001; Debnath and Pande, 2003; Li et al., 2017). For digestive system and other adverse reactions, no statistical difference was found. Four RCTs (Liu et al., 2014; Zhai et al., 2014; Faritus et al., 2015; Li et al., 2017) comparing midazolam oral solution with dexmedetomidine, with 260 children included, indicated that the incidence of adverse reactions in midazolam oral solution was higher [RR = 7.22, 95% CI (2.85, 18.28), *I*^2^ = 0%, *P* < 0.05]. For the incidence of adverse effects of the mental system, midazolam oral solution was higher [RR = 12.00, 95% CI (2.93, 49.23), *I*^2^ = 0%, *P* < 0.05] (Liu et al., 2014; Zhai et al., 2014; Li et al., 2017). For digestive system, respiratory system, and other adverse reactions, no statistical difference was found. Three RCTs (Luhmann et al., 2001; Keidan et al., 2005; You et al., 2018) comparing midazolam oral solution with N_2_O, with 209 children included, indicated no significant difference [RR = 1.54, 95% CI (0.22, 10.57), *I*^2^ = 58%, *P* = 0.66]. But the incidence of mental system adverse effects in midazolam oral solution was higher [RR = 6.78, 95% CI (1.29, 36.53), *I*^2^ = 36%, *P* = 0.02] (Funk et al., 2000; You et al., 2018).

Information on the adverse reactions of midazolam in various countries or regions was as follows. The Food and Drug Administration (Food Drug Administration, 2016) revised the midazolam syrup instructions by supplementing the risk information for children's medications, indicating that oral midazolam carried a higher risk of serious life-threatening adverse events for children with congenital heart disease and pulmonary hypertension and required children to start with a low dose to avoid breathing problems. The drug instructions of the Sweden Medical Products Agency and the Ireland Medicines Board showed that airway obstruction and hypoventilation should be avoided when using midazolam in pediatric patients <6 months, especially for children with cardiovascular disease (Health Products Regulatory Authority., 2019; Medical Products Agency, 2019). Regarding the use of midazolam in children, the Canada Vigilance Adverse Reaction Online Database showed that from 1 January 1965 to 2 August 2018, a total of 22 adverse reactions in children were recorded, mainly characterized by ataxia, loss of appetite, erythema, macula rash, myoclonus, and hyperhidrosis (Health Canada, 2018). New Zealand's Medicines and Medical Devices Safety Authority Database showed that from 1 January 2000 to 2 August 2018, two cases of suspected childhood adverse reactions were observed, mainly characterized by urticaria, agitation, and disorientation (Medicines Medical Devices Safety Authority, 2018). In February 1998, the Australian Bulletin on Adverse Drug Reactions (vol. 17, no. 1) showed that 31 cases of adverse reactions occurred, including 18 cases of agitation, 11 cases of aggression, 9 cases of abnormal crying, 7 cases of hallucinations, and 3 cases of emotional instability, and 20 of them occurred in children aged 11 years or younger (The therapeutic Goods Administration, 2018). There were no reports of midazolam adverse reactions in the WHO, China National Center for ADR Monitoring, and other searched adverse drug monitoring centers.

## Discussion

### Summary of Main Findings

Certain examinations and treatments for children require sedation and hypnosis. The success rate of sedation and hypnosis and the duration of sedative hypnosis are widely used outcome measures. Both its therapeutic effects and adverse reactions are due to its neuronal inhibitory pathways by affecting the gamma-aminobutyric acid (GABA) receptor (Jacqz-Aigrain and Burtin, 1996). We systematically reviewed the efficacy and safety of midazolam oral solution for sedative hypnosis and antianxiety in children.

No statistically significant difference in the efficacy and adverse effects of midazolam for oral solution and injection was found. Neither was there a statistically significant difference in the incidence of adverse reactions between the midazolam oral solution and the blank or placebo group. There was inconsistent evidence that oral midazolam decreased anxiety during procedures compared with placebo. Oral solution has the advantages of convenience, non-invasiveness, safety, and economy (European Medicine Agency, 2006). Midazolam was more effective than other benzodiazepines (hydroxyzine and diazepam). Midazolam is preferred over other benzodiazepines because of its water solubility and rapid clearance.

The systematic review showed that the midazolam oral solution had a lower sedation success rate than chloral hydrate, consistent with the results of the previous systematic review (Mataftsi et al., 2017), but the evidence quality was low, and the chloral hydrate group dose was higher than the regular clinical dose. The overall incidence of adverse effects was comparable. Three RCTs showed that midazolam oral solutions had a higher incidence of mental adverse events [RR = 5.67, 95% CI (3.54, 9.09), *I*^2^ = 0%, *P* < 0.05] and a lower incidence in the digestive system [RR = 0.24, 95% CI (0.07, 0.78), *I*^2^ = 34%, *P* = 0.02]. One cohort study showed no statistically significant difference in each system, but the ratio of experimental groups compared with the control group was approximately 1:20, which could lead to false-negative results (Schmidt et al., 2018). Chloral hydrate is now commonly used in clinical practice for sedation and hypnosis for diagnosis or treatment. It is strangely odorous, so the child's compliance of oral solution is poor, which makes it easy to cough and even suffocate. The rectal administration of chloral hydrate is easy to stimulate the intestinal wall, and the administration process is troublesome. The active metabolites of chloral hydrate may cause long-term sedation and a narrow therapeutic index (Abbas et al., 1996). Both chloral hydrate and midazolam are recommended by the American Institute for Safe Medication Practices as a high-risk drug (Institute for Safe Medication Practices, 2019). The total adverse reaction rate of midazolam oral solution was not statistically different from that of chloral hydrate. Therefore, clinical applications of chloral hydrate and midazolam oral solution should be done with caution.

The sedative and hypnotic success rate of oral solution of midazolam was equivalent to that of dexmedetomidine. The incidence of neuropsychiatric adverse reactions in midazolam oral solution is higher [RR = 12.00, 95% CI (2.93, 49.23), *I*^2^ = 0%, *P* < 0.05]. A systematic review (Zhang et al., 2014) showed that dexmedetomidine reduced the risk of agitation or paralysis, chills, systolic blood pressure, and heart rate in children compared with midazolam. The bioavailability of dexmedetomidine oral sedation is poor, about 16%, and its nasal mucosa absorption is more stable (Uusalo et al., 2019). The pharmacokinetics of dexmedetomidine is highly characterized by individual differences, especially in the intensive care unit population (Weerink et al., 2017).

Our evidence suggests that clinical midazolam oral solutions are used in children for sedative hypnosis at doses ranging from 0.25 to 1.0 mg/kg. Our review showed that the time for children to fall asleep was longer in the dose of (0.25–0.5) mg/kg compared to (0.5–1.0) mg/kg, and the two groups were equivalent in relieving anxiety and parental success rate. And the incidence of adverse reactions was similar [10% for (0.25–0.5) mg/kg and 9% (0.5–1.0) mg/kg]. These results suggest that the oral solution of 0.25–1.0 mg/kg of midazolam is effective and safe, and the safe and effective dose of midazolam in the pediatric surgery outdoor anesthesia/sedation expert consensus (2017) is 0.50–0.75 mg/kg, so they are consistent to some extent (Chinese Medical Association, 2011).

We performed a subgroup analysis comparing midazolam with other drugs, based on different doses of midazolam. The doses of midazolam oral solution were divided into three dose groups: (0–0.25), (0.25–0.5), and (0.5–1.0) mg/kg. The results did not differ from those before grouping. This may be the result of the insufficient numbers of included studies.

### Limitations

This systematic review had some limitations. First, the literature included in this study is limited to Chinese and English, and thus, there may be language bias. Second, 77 of the 89 RCTs did not clearly describe the allocation concealment. If researchers and authors document their experimental methods in future clinical trials and publications in detail, readers and reviewers can better understand the true content of the study. Third, of these 89 RCTs, the smallest sample size is 10 (median 60). A small sample size results in a less authentic result. We look forward to a larger population and long-term data to fully assess the efficacy and risk.

## Conclusion

Limited evidence suggests that midazolam is effective and safe prior to a diagnosis or treatment procedure for sedation and hypnosis in children. Available evidence suggests that midazolam oral solution is as good as midazolam injection and dexmedetomidine and is better than ketamine. The success rate of sedation and hypnosis of midazolam oral solution was lower than that of chloral hydrate. Neuropsychiatric adverse reactions of midazolam are higher than those of chloral hydrate and dexmedetomidine. Digestive system adverse reactions of midazolam are lower than those of chloral hydrate.

## Author Contributions

LZh, ZC, LZe, LH, DY, XC, and PX conceived and designed the review. XC, PX, FQ, XJ, YW, and ML reviewed the literature and extracted data. XC and ZC wrote the manuscript.

### Conflict of Interest

The authors declare that the research was conducted in the absence of any commercial or financial relationships that could be construed as a potential conflict of interest.
