# Supplementary material for: Efficacy and Safety of Midazolam Oral Solution for Sedative Hypnosis and Anti-anxiety in Children: A Systematic Review and Meta-Analysis
Source: Front Pharmacol. 2020 Mar 18;11:225. doi: 10.3389/fphar.2020.00225 (PMC7093581; doi:10.3389/fphar.2020.00225)
Supplement: Supplementary file 1 [file Data_Sheet_1.docx]

***Supplementary Material***

**Supplementary Table 1**

**Table 1**: Characteristics of included studies

| **Study** | **Sample** | **Region** | **Interventions** | **Characteristics of midazolam** | | **Age (years)** | **Weight (kg)** | **Procedure type** | **Study type** | **Outcome measure** |
| --- | --- | --- | --- | --- | --- | --- | --- | --- | --- | --- |
|  |  |  |  | Dosage form | Dose |  |  |  |  |  |
| Halim M Hennes 1990 | 55 | America | T:Oral midazolam 0.2 mg/kg (n=30) C:Placebo (n=25) | - | - | T:2.92±0.08 C:2.33±0.11 | - | Invasive | RCT | - |
| K.A.Payne 1991 | 135 | - | T:Oral midazolam 0.45 mg/kg (n=34) C1:Blank (n=33) C2:Oral isobutyrazine tartrate 2 mg/kg + Methadone 0.1 mg/kg + Droperidol 0.15 mg/kg (n=35) C3: Intramuscular injection of midazolam 0.15 mg/kg (n=33) | - | - | T:5.5±3.6 C1:5.7±3.7 C2:5.5±3.5 C3:6.4±3.8 | T:16.1±7.0 C1:15.8±6.5 C2:16.2±7.2 C3:16.7±6.1 | Invasive | RCT | Success rate of sedation and hypnosis |
| Mcmillan 1992 | 80 | - | T1:Oral midazolam 0.5 mg/kg (n=20) T2:Oral midazolam 0.75 mg/kg (n=20) T3:Oral midazolam 1.0 mg/kg (n=20) C:Placebo (n=20) | - | - | T1:3.7±1.4 T2:3.4±1.5 T3:3.4±1.5 C:3.7±1.3 | T1:16.4±3.5 T2:11.7±3.0 T3:15.7±3.5 C:15.3±3.4 | Invasive | RCT | Degree of calm, anxiety scores |
| Parnis 1992 | 200 | Australia | T:Oral midazolam 0.5 mg/kg (n=29) C:Placebo (n=25) | - | - | - | - | Invasive | RCT | - |
| Weldon 1992 | 54 | America | T:Oral midazolam 0.5 mg/kg (n=29) C:Placebo (n=25) | - | - | T:3.5±2.0 C:3.5±2.1 | T:16.0±5.5 C:15.8±6.4 | Invasive | RCT | Adverse reactions |
| Vetter 1993 | 75 | America | T:Oral midazolam 0.6 mg/kg (n=25) C1: Oral diazepam 0.3 mg/kg (n=25) C2:Placebo (n=25) | - | - | 1-6 | - | Invasive | RCT | - |
| AIderson 1994 | 40 | - | T:Oral midazolam 0.5 mg/kg (n=20) C:Oral ketamine 5 mg/kg (n=20) | - | - | T:3.3±1.1 C:3.4±1.3 | T:15.8±3.0 C:14.9±1.3 | Invasive | RCT | - |
| Thomas S 1994 | 31 | - | T:Oral midazolam 0.3 mg/kg (n=16) C:Oral midazolam 0.5 mg/kg (n=15) | Injection | - | T:4-17 C:3-18 | - | Invasive | RCT | Success rate of sedation and hypnosis |
| Lyons1995 | 78 | - | T:Oral midazolam 0.5 mg/kg (n=26) C1: Rectal thiopental 35 mg/kg (n=25) C2:Blank (n=24) | - | - | T:3.13±0.10 C1:2.38±0.10 C2:2.74±0.11 | T:15.2±2.89 C1:13.8±2.48 C2:13.8±3.14 | Invasive | RCT | Anxiety scores |
| Cray 1996 | 44 | - | T:Oral midazolam 0.5 mg/kg (n=26) C:Placebo (n=18) | Injection | - | T:4.5 C:4 | T:18 C:19 | Invasive | RCT | Anxiety scores |
| Daniel A 1996 | 23 | - | T:Oral midazolam 0.5 mg/kg (n=23) C:Oral chloral hydrate 50 mg/kg (n=23) | - | -- | 6.8 | - | Invasive | RCT | Degree of calm |
| Riva 1997 | 105 | Urμguay | T:Oral midazolam 0.75 mg/kg (n=36) C1:Blank (n=29) C2:Placebo (n=40) | - | - | T:6.7±2.3 C1:6.3±2.3 C2:5.8±2 | T:24.5±7.0 C1:24.7±8.4 C2:22.9±6.2 | Invasive | RCT | - |
| V.Mitchell 1997 | 85 | - | T:Oral midazolam 0.5 mg/kg (n=28) C1:Oral isobutyrazine 2 mg/kg (n=28) C2:Placebo (n=29) | - | - | T:5.43±1.6 C1:5.58±1.89 C2:5.34±1.92 | T:20.74±4.78 C1:20.99±4.72 C2:20.79±5.63 | Invasive | RCT | Degree of calm, adverse reactions |
| Chris A. Liacouras 1998 | 123 | America | T:Oral midazolam 0.2 mg/kg (n=61) C:Placebo (n=62) | -- |  | 7.75±4.46 | - | Noninvasive | RCT | Success rate of sedation and hypnosis |
| F C Davies 1998 | 50 | - | T1:Oral midazolam 0.5 mg/kg (n=9) T2:Oral midazolam 0.2 mg/kg (n=41) | - | - | - | - | Invasive | RCT | - |
| Dagostino 2000 | 33 | - | T:Oral midazolam 0.5 mg/kg (n=22) C:Oral chloral hydrate 75 mg/kg (n=11) | - | -- | T:2.50±0.17  C:2.67±0.15 | T:11.9±5.6 C:12.9±4.7 | Noninvasive | RCT | Success rate of sedation and hypnosis, duration of sedative hypnosis (min), adverse reactions |
| W. Funk 2000 | 113 | - | T:Oral midazolam 0.5 mg/kg (n=38) C1:Oral ketamine 6 mg/kg (n=36) C2:Oral midazolam 0.5 mg/kg and ketamine 3 mg/kg (n=39) |  | - | T:5.7 C1:5.8 C2:5.7 | T:19.6±4.7 C1:20.8±4.8 C2:19.6±4.6 | Invasive | RCT | - |
| Luhmann 2001 | 152 | - | T:Oral midazolam 0.5 mg/kg (n=51) C1:Inhaled nitrous oxide (50% N_2_O, 50% O_2_, speed 6-10 L/min (n=51) C2:Blank (n=50) | - | - | - | - | Invasive | RCT | Adverse reactions |
| Wheeler 2001 | 40 | America | T:Oral midazolam 0.5 mg/kg (n=25) C:Oral chloral hydrate 75 mg/kg (n=15) | Injection | - | T:1.23±0.08 C:1.11±0.06 | T:8.96±3.17 C:9.21±2.79 | Noninvasive | RCT | Success rate of sedation and hypnosis, time to fall asleep (min), duration of sedative hypnosis (min) |
| Younge 2001 | 57 | UK | T:Oral midazolam 0.7 mg/kg (n=28) C:Oral ketamine 10 mg/kg (n=29) | - | - | 4.1 | - | Invasive | RCT | Adverse reactions |
| Charles J. Cote 2002 | 397 | - | T1:Oral midazolam 0.25 mg/kg (n=132) T2:Oral midazolam 0.5 mg/kg (n=132) T3:Oral midazolam 1.0 mg/kg (n=133) | - | - | T:4.7±3.8 C1:4.5±3.6 C2:4.6±3.9 | T:20.2±13.0 C1:19.4±11.7 C2:20.0±13.7 | Invasive | RCT | - |
| Ian J. everitt 2002 | 129 | Australia | T:Oral midazolam 1.0 mg/kg (n=45) C1:Oral diazepam 0.5 mg/kg (n=42) C2:Intranasal midazolam 0.4 mg/kg (n=42) | - | - | 1-5 | - | Invasive | RCT | - |
| Brosius KK 2002 | 50 | - | T:Oral midazolam 20 mg (n=25) C:Placebo (n=25) | - | - | T:14.4±2.4 C:14.4±2.1 | T:60.9±13.9 C:57.6±12.6 | Invasive | RCT | Success rate of sedation and hypnosis |
| Wilson 2002 (Crossover) | 26 | UK | T:Oral midazolam 0.5 mg/kg (n=26) C:Inhaled nitrous oxide (n=26) | - | - | 12.5 (10-16) | - | Invasive | RCT | Success rate of sedation and hypnosis |
| Mcerlean2003 | 51 | - | T:Oral midazolam 0.5 mg/kg (n=24) C:Placebo (n=27) | - | - | 0.75-6.00 | - | Invasive | RCT | - |
| S.N.Wildt 2003 | 24 | - | T:Oral midazolam 0.1 mg/kg (n=11) C:Intravenous infusion of midazolam 0.1 mg/kg (n=20) | Injection | - | T:6.0±2.7 day C:6.5±3.1 day | - | Invasive | RCT | - |
| Singh 2003 | 90 | India | T:Oral midazolam 0.5 mg/kg (n=30) C1:Oral Triclofos70 mg/kg (n=30) C2:Oral promethazine 1.2 mg/kg (n=30) | - | - | - | - | Invasive | RCT | Degree of calm, time to fall asleep (min), duration of sedative hypnosis (min) |
| Suranjit Debnath 2003 | 60 | - | T:Oral midazolam 0.5 mg/kg (n=30) C:Oral ketamine 6 mg/kg (n=30) | - | - | 1-10 | - | Invasive | RCT | Degree of calm, anxiety scores, time to fall asleep (min), adverse reactions |
| Tamura 2003 | 51 | Japan | T:Oral midazolam 5 mg (n=24) C:Oral fentanyl 0.1 mg (n=27) | - | - | T:4.92±0.14 C:4.92±0.15 | T:18±4.1 C:18±3.7 | Invasive | RCT | - |
| Brad fine 2004 | 45 | - | T:Oral midazolam 0.5 mg/kg (n=15) C1:Intramuscular injection of midazolam 0.2 mg/kg (n=15) C2: Subcutaneous injection of midazolam 0.2 mg/kg (n=15) | - | - | T:3.0±1.2 C1:2.6±1.0 C2:2.3±1.2 | T:14±3 C1:13±3 C2:17±3 | Invasive | RCT | - |
| Toshinori Horiuchi 2004 | 55 | Japan | T:Oral midazolam 0.5 mg/kg (n=28) C:Oral ketamine 50 mg (n=27) | - | - | T:4.2±1.3 C:4.3±1.1 | T:17±4 C1:7±3 | Invasive | RCT | - |
| Wan kuo 2005 | 40 | China | T:Oral midazolam 0.5 mg/kg (n=21) C:Placebo (n=19) | - | - | 7.3 (5-10) | 22.9 (16-32) | Invasive | RCT | Degree of calm |
| Ilan keidan 2005 | 47 | America | T:Oral midazolam 0.5 mg/kg (n=24) C:Inhaled nitrous oxide (n=23) | - | - | T:6±2.5 C:7±3.0 | - | Noninvasive | RCT | Anxiety scores, adverse reactions |
| Mishra2005 | 100 | - | T1:Oral midazolam 0.5 mg/kg (n=25) T2:Oral midazolam 0.75 mg/kg (n=25) T3:Oral midazolam 1.0 mg/kg (n=25) C:Placebo (n=25) | Injection | - | T1:3.48±2.20 T2:3.61±2.27 T3:3.96±2.04 C:3.46±2.16 | T1:13.5±7.5 T2:14.0±8.5 T3:13.5±7.0 C:14.5±6.5 | Invasive | RCT | Success rate of parental separation, degree of calm, adverse reactions |
| Singh 2005 | 60 | India | T:Oral midazolam 0.5 mg/kg (n=30) C:Oral butorphanol 0.2 mg/kg (n=30) | - | - | T:4.9±0.6 C:5.1±0.5 | T:11.60±3.6 C:12.20±2.9 | Invasive | RCT | Degree of calm, time to fall asleep (min) |
| Tamaporn P, 2005 | 13 | - | T:Oral midazolam 0.5 mg/kg (n=13) C:Oral diazepam 0.3 mg/kg (n=13) | - | - | 5.8-14.7 | - | Invasive | RCT | Success rate of sedation and hypnosis |
| Y-C. P. Arai 2005 | 42 | - | T:Oral midazolam 0.5 mg/kg (n=14) C1:Oral midazolam 0.25 mg/kg and diazepam 0.25 mg/kg (n=14)  C2:Blank (n=14) | - | - | T:4 C1:5 C2:4 | T:18 C1:17 C2:15 | Invasive | RCT | - |
| Liu jun 2006 | 40 | China | T:Oral midazolam 0.5 mg/kg (n=20) C:Intramuscular injection of midazolam 0.1 mg/kg (n=20) | - | 5 mg/ml | - | - | Invasive | RCT | - |
| Bandana Koirala 2006 | 100 | - | T:Oral midazolam 0.5 mg/kg (n=20) C1:Oral ketamine 5 mg/kg (n=20) C2: Oral pyrazol 0.4 mg/kg (n=20) C3:Oral midazolam 0.4 mg/kg and Ketamine3 mg/kg (n=20) C4:Oral midazolam 0.5 mg/kg and Tramado 2 mg/kg (n=20) | - | - | 2-9 | - | Invasive | RCT | - |
| K. E. Wilson 2006 | 40 | - | T:Oral midazolam 0.3 mg/kg (n=40) C:Inhaled nitrous oxide (n=40) | - | - | 5-10 | - | - | RCT | - |
| Selman Vefa Yildirim 2006 | 80 | - | T:Oral midazolam 0.4 mg/kg (n=30) C1:Intravenous infusion of midazolam 0.2 mg/kg (n=30) C2:Placebo (n=20) | - | - | T:0.5-2.5 C1:0.5-3.0 C2:0.5-2.3 | - | Noninvasive | RCT | Success rate of sedation and hypnosis, adverse reactions |
| Andre 2007 | 42 | - | T:Oral midazolam 0.5 mg/kg (n=22) C:Dexmedetomidine transdermal patch 1 μg/kg (n=20) | - | - | T:9 C1:10 C2:8 | T:33 C1:34.9 C2:26 | - | RCT | - |
| Mccluskey 2007 | 51 | - | T:Oral midazolam 0.5 mg/kg (n=24) C:Placebo (n=27) | - | - | T:5.4±2.5,  C:4.8±2.5 | T:20.2±6.3 C:19.8±6.9 | Invasive | RCT |  |
| Simonetta Tesoro 2007 | 53 | - | T:Oral midazolam 0.9 mg/kg C:Oral triazolam 0.5 mg/kg | - | - | - | - | Noninvasive | RCT | Time to fall asleep (min), duration of sedative hypnosis (min) |
| Damle S.G 2008 | 20 | India | T:Oral midazolam 0.5 mg/kg (n=10) C:Oral ketamine 5 mg/kg (n=10) | - | - | 2-6 | - | Invasive | RCT | Success rate of sedation and hypnosis |
| Chen li 2009 | 60 | China | T1:Oral midazolam 0.2 mg/kg (n=20) T2:Oral midazolam 0.25 mg/kg (n=20) T3:Oral midazolam 0.3 mg/kg (n=20) | - | - | T:2.9±0.8 C1:2.7±0.7 C2:2.6±0.8 | T:14±2 C1:14±3 C2:13±2 | Invasive | RCT | Anxiety scores |
| Mark D. Talon 2009 | 100 | - | T:Oral midazolam 0.5 mg/kg (n=50) C:Nasal drop dexmedetomidine 2 μg/kg (n=50) | - | - | T:10.72±4.53 C:9.58±4.37 | T:35.51±16.79 C:39.88±9.5 | - | RCT | Success rate of sedation and hypnosis |
| Kajal Jain 2010 | 92 | - | T:Oral midazolam 0.5 mg/kg (n=29) C1:Oral midazolam 0.25 mg/kg and ketamine 1 mg/kg (n=31) C2:Placebo (n=32) | - | - | T:3.2±1.12 C1:3.3±1.16 C2:3.4±1.27 | T:12.4±2.4 C1:13.6±3.1 C2:13.8±3.4 | Noninvasive | RCT | Degree of calm |
| Mandana Rafeey 2010 | 61 | Iran | T:Oral midazolam 0.5 mg/kg (n=30) C:Intravenous infusion of midazolam 0.05-0.1 mg/kg (n=31) | - | - | T:6.30±2.91 C:7.98±3.71 | - | Noninvasive | RCT | Duration of sedative hypnosis (min) |
| Peter Templeton 2010 | 42 | - | T:Oral midazolam 0.2 mg/kg (n=20) C:Placebo (n=22) | - | - | T:7.2±1.7 C:6.9±1.9 | T:26.8±7.5 C:25.7±7.1 | Invasive | RCT | Anxiety scores |
| Genoa G. Ferguson 2011 | 44 | America | T:Oral midazolam 0.5 mg/kg (n=22) C:Placebo (n=22) | - | - | T:3.59 C:3.45 | - | Noninvasive | RCT | Anxiety scores |
| M. Ghali 2011 | 120 | - | T:Oral midazolam 0.5 mg/kg (n=60) C:Nasal drop dexmedetomidine 1 μg/kg (n=60) | - | - | T:8.1±2.3 C:8.2±1.4 | T:17.9±5.89 C:18.40±4.74 | Invasive | RCT | Success rate of parental separation, degree of calm, anxiety scores |
| Mehrdad Shoroghi 2011 | 90 | Iran | T1:Oral midazolam 0.5 mg/kg (n=30) T2:Oral midazolam 1.0 mg/kg (n=30) C:Placebo (n=30) | - | - | T1:5.1±1.4 T2:4.4±1.5 C:4.7±1.7 | T1:19.1±3.5 T2:21.0±2.9 C:22.3±3.4 | Noninvasive | RCT | Adverse reactions |
| Mostafa Somri 2011 | 90 | - | T1:Oral midazolam 0.5 mg/kg (n=30) T2:Oral midazolam 0.75 mg/kg (n=30) T3:Oral midazolam 1.0 mg/kg (n=30) | - | - | T:5.6±1.85 C1:5.6±1.67 C2:6.2±2.00 | T:19.2±3.68 C1:19.7±3.38 C2:20.3±3.65 | Invasive | RCT | Degree of calm, time to fall asleep (min) |
| Mountain B W 2011 | 41 | 27 white, 9 African American, 5 Hispanic | T:Oral midazolam 0.5 mg/kg (n=19) C:Oral dexmedetomidine 4 μg /kg (n=2) | - | - | 4 | - | Invasive | RCT | Success rate of mask acceptance, success rate of parental separation |
| Özcengiz D2011 | 100 | - | T:Oral midazolam 0.5 mg/kg (n=25) C1:Placebo (n=25) C2:Oral dexmedetomidine 2.5 μg/kg (n=25) C3: Melatonin 0.1 mg/kg (n=25) | - | - | 3-9 | - | Invasive | RCT | - |
| Shabbir A 2011 (Self-controlled) | 12 | India | T:Oral midazolam 0.5 mg/kg (n=12) C:Triclofos 70 mg/kg (n=12) | - | - | 3-9 | - | Invasive | RCT | - |
| Hua li 2012 | 30 | China | T:Oral midazolam 0.5 mg/kg (n=10) C1:Intramuscular injection of ketamine 2 mg/kg (n=10) C2:Fentanyl nasal drops 10μg/kg (n=10) | - | - | 1-3 | 10-19 | Invasive | RCT | Degree of calm |
| Chandni Sinha 2012 | 60 | - | T:Oral midazolam 0.5 mg/kg (n=30) C:Oral butorphanol 0.2 mg/kg (n=30) | - | - | T:6.90±2.99 C:6.23±2.59 | T:19.77±6.15 C:20.00±7.25 | Invasive | RCT | Degree of calm |
| Wang wu 2013 | 120 | China | T1:Oral midazolam 0.5 mg/kg (n=30) T2:Oral midazolam 0.25 mg/kg (n=30) T3:Oral midazolam 0.75 mg/kg (n=30) C:Placebo (n=30) | - | - | T:5.8±2.7 C1:6.1±2.7 C2:6.0±2.9 C3:6.3±2.5 | T:25±5 C1:26±5 C2:27±6 C3:25±4 | Invasive | RCT | - |
| Ashrafi, 2013 | 198 | Iran | T:Oral midazolam 0.5 mg/kg (n=100) C: Oral 5% chloral hydrate 1 ml/kg (n=98) | - | - | T:4 (0.17-9) C:4 (0.25-10) | - | Noninvasive | RCT | Adverse reactions |
| Hojjat Derakhshanfar 2013 | 160 | - | T:Oral midazolam 0.5 mg/kg (n=80) C:Oral chloral hydrate 80 mg/kg (n=80) | - | - | T:3.6±2.6,  C:3.4±1.9 | T:18.1±4.2 C:16.8±5.1 | Invasive | RCT | Success rate of sedation and hypnosis, degree of calm, time to fall asleep (min), duration of sedative hypnosis (min), adverse reactions |
| Isabelita Duarte Azevedo 2013 | 10 | - | T:Oral midazolam 0.3 mg/kg (n=31) C:Placebo (n=29) | - | - | 3.7 | - | Invasive | RCT | Degree of calm |
| Thakurta 2013 | 70 | India | T:Oral midazolam 0.5 mg/kg (n=35) C:Oral ketamine 5 mg/kg (n=35) | - | - | T:4.67±2.2 C:5.43±1.94 | T:14.76±4.84 C:16.43±4.24 | Invasive | RCT | Degree of calm |
| Tyagi 2013 | 40 | India | T:Oral midazolam 0.5 mg/kg (n=35) C:Oral ketamine 5 mg/kg (n=35) | -- | - | 2-10 | - | Invasive | RCT | - |
| Liu chun 2014 | 60 | China | T:Oral midazolam 0.5 mg/kg (n=30) C:Oral dexmedetomidine 4 μg/kg (n=30) | - | - | T:2.6±1.54 C:2.4±1.21 | T:15.3±4.24 C:14.2±3.72 | - | RCT | Degree of calm, adverse reactions |
| Zhai hongqin 2014 | 100 | China | T:Oral midazolam 0.5 mg/kg (n=50) C:Nasal drop dexmedetomidine 1 μg/kg (n=50) | - | - | T:5.4±2.0 C:5.8±2.1 | - | - | RCT | Adverse reactions |
| Abhishek K Phadke 2014 | 272 | - | T:Oral midazolam 0.5 mg/kg (n=136) C:Intravenous infusion of midazolam 0.15 mg/kg (n=136) | - | - | T:4.5 C:5.1 | - | Both | RCT | Degree of calm |
| Azarfar2014 | 84 | - | T:Oral midazolam 0.5 mg/kg (n=42) C:Blank (n=42) | - | - | 6.8±1.85 | - | Noninvasive | RCT | Success rate of sedation and hypnosis |
| B. Linares Segovia, 2014 | 108 | Mexico | T:Oral midazolam 0.5 mg/kg (n=56) C:Nasal drop dexmedetomidine 1 μg/kg (n=52) | - | - | T:4 (3-5) C:4 (3-5) | T:17.4±7.12 C:16.0±5.6 | Invasive | RCT | - |
| Savla 2014 | 52 | India | T:Oral midazolam 0.5 mg/kg (n=15) C1::Nasal drop dexmedetomidine 2 μg/kg (n=119) C2:Placebo (n=18) | Syrup | 2 mg/ml | T:4 (1–8) C1:3 (1–6) C2:4 (1–6) | T:14 (4–21) C1:12 (6–22) C2:13 (4–22) | Invasive | RCT | - |
| Sujata Chaudhary 2014 | 60 | India | T:Oral midazolam 0.5 mg/kg (n=20) C1:Oral triclofos 75 mg/kg (n=20) C2: Oral hydroxyzine 0.5 mg/kg (n=20) | - | - | T:4.90±2.05 C1:4.85±1.98 C2:4.90±1.97 | T:15.75±5.38 C1:13.95±4.21 C2:16.80±5.62 | Invasive | RCT | Success rate of mask acceptance |
| Suman Arora, 2014 | 56 | India | T:Oral midazolam 0.5 mg/kg (n=29) C:Oral dexmedetomidine 4 μg/kg (n=27) | - | - | T:2.2±1.2 C1:3.0±1.0 C2:3.0±1.0 | T:12.4±3.4 C1:13.9±3.4 C2:12.9±3.2 | Invasive | RCT | Success rate of sedation and hypnosis, success rate of mask acceptance, success rate of parental separation |
| faritus2015 | 60 | - | T:Oral midazolam 0.5 mg/kg (n=30) C:Oral dexmedetomidine 2 μg/kg (n=30) | - | - | T:3.63±1.81,  C:4.28±2.22 | T:13.80±4.49,  C:14.33±4.68 | Invasive | RCT | Degree of calm, adverse reactions |
| Belen FB 2015 | 47 | - | T:Oral midazolam 0.2 mg/kg (n=24) C:Placebo (n=23) | - | - | 9.8±5.8 (2-18) | - | Invasive | RCT | - |
| Hisham Y 2015 | 78 | - | T:Oral midazolam 0.5 mg/kg (n=39) C:Placebo (n=39) | - | - | T:5.4±1.4,  C:5.6±1.2 | T:19.5±2.1 C:19±3.1 | Invasive | RCT | Success rate of parental separation |
| Song yunan 2016 | 442 | China | T:Oral midazolam 0.5 mg/kg (n=200) C:Blank (n=242) | - | - | T:＜0.67 C1:6.1±2.7 C2:3-5 C3:5-7 C4:＞7 | - | Invasive | RCT | - |
| Ahmad Khodadad 2016 | 120 | - | T:Oral midazolam 0.5 mg/kg (n=59) C:Intravenous infusion of midazolam 0.1 mg/kg (n=60) | - | - | 6.8±3.3 | - | Noninvasive | RCT | Success rate of sedation and hypnosis, success rate of parental separation, degree of calm, time to fall asleep (min), adverse reactions |
| Babita Ghai 2016 | 59 | - | T:Oral midazolam 0.5 mg/kg (n=29) C:Nasal drop dexmedetomidine 2.5 mcg/kg (n=30) | - | - | T:3.1±1.3,  C:3.8±2.1 | T:12.6±2.7 C:13.0±3.8 | Noninvasive | RCT | Success rate of sedation and hypnosis |
| Omar Hijazi 2016 | 286 | - | T:Oral midazolam 0.5 mg/kg (n=142) C:Oral chloral hydrate 75 mg/kg (n=144) | Injection | - | T:2.18±0.16 C:2.21±0.14 | T:11.90±5.84 C:11.15±3.76 | Invasive | RCT | Success rate of sedation and hypnosis, time to fall asleep (min), duration of sedative hypnosis (min), adverse reactions |
| Orit Rubinstein 2016 | 68 | Israel | T:Oral midazolam 0.7 mg/kg (n=31) C:Oral ketamine 5 mg/kg (n=37) | - | - | T:4.50±2.12 C:5.57±2.07 | T:18.51±6.64 C:19.91±6.36 | Invasive | RCT | Success rate of sedation and hypnosis, degree of calm, time to fall asleep (min) |
| Li min 2017 | 60 | China | T:Oral midazolam 0.5 mg/kg (n=18) C1:Oral ketamine 5 mg/kg (n=20) C2:Oral dexmedetomidine 5μg/kg (n=20) | - | 5 mg/ml | - | - | Invasive | RCT | Time to fall asleep (min), adverse reactions |
| Forod Salehi 2017 | 68 | Iran | T:Oral midazolam 0.2 mg/kg (n=34) C:Oral chloral hydrate 50 mg/kg (n=34) | - | - | T1:0.43±0.13 C:1.19±0.10 | - | Noninvasive | RCT | Time to fall asleep (min), duration of sedative hypnosis (min), adverse reactions |
| M Kavya Prabhu 2017 | 90 | - | T:Oral midazolam 0.5 mg/kg (n=45) C:Oral dexmedetomidine 2 μg/kg (n=45) | - | - | T:6.53±2.27 C:6.47±2.19 | T:18.09±5.29 C:18.29±4.02 | - | RCT | - |
| Sarika Kumari 2017 | 90 | India | T:Oral midazolam 0.5 mg/kg (n=30) C1:Oral dexmedetomidine 4 μg/kg (n=30) C2: Oral clonidine 4 μg/kg (n=30) | Injection | 5 mg/ml | T:6.63±2.8 C1:7.9±3.21 C2:7.45±2.91 | T:19.95±6.93 C1:20.27±8.59 C2:19.07±6.76 | Invasive | RCT | Success rate of sedation and hypnosis, success rate of mask acceptance, success rate of parental separation, degree of calm, anxiety scores |
| You jie 2018 | 91 | China | T:Oral midazolam 0.5-0.75 mg/kg (n=30) C1:Inhaled nitrous oxide (n=30) C3:Oral midazolam 0.5 mg/kg + Inhaled nitrous oxide (n=31) | Tablet | 7.5 mg/ tablet | T:9.10±4.51 C1:8.90±5.33 C2:9.23±4.85 | - | - | RCT | Success rate of sedation and hypnosis, adverse reactions |
| Kolathu 2018 | 60 | India | T:Oral midazolam 0.5 mg/kg (n=30) C: Triclofos 100 mg/kg (n=30) | - | - | - | - | Invasive | RCT | Success rate of sedation and hypnosis, success rate of mask acceptance, success rate of parental separation |
| Madhuri S Kurdit 2018 | 100 | - | T:Oral midazolam 0.5 mg/kg (n=25) C1:Oral melatonin 0.5 mg/kg (n=25) C2:Oral melatonin 0.75 mg/kg (n=25) C3:Placebo (n=25) | - | - | T1:1.04±2.92 C1:10.72±2.41 C2:11.04±2.70 C3:9.16±2.62 | T:32.28±8.34 C1:30.28±5.78 C2:30.32±7.64 C3:23.36±7.34 | Invasive | RCT | Anxiety scores |
| Reyhaneh Faghihian 2018 | 132 | Iran | T:Oral midazolam 0.5 mg/kg (n=44) C1:Oral melatonin 0.5 mg/kg (n=44) C2:Placebo (n=44) | - | 15 mg/3ml | T:4.3±0.96 C1:3.9±0..98 C2:4.1±1.01 | - | Invasive | RCT | - |
| Jack S 1995 | 122 | America | T:Oral midazolam 0.6 mg/kg (n=97) C:Blank (n=25) | - | 5 mg/ml | T:4.4 (1.92-9) C:4.6 (3-8) | - | Invasive | Cohort study | Adverse reactions |
| Jason J. Horgesheimer 2001 | 106 | - | T:Oral midazolam 0.5 mg/kg (n=50) C:Blank (n=56) | - | - | T:3.8±1.7 C:3.7±1.5 | T:15.6±3.3 C:15.4±3.8 | - | Cohort study | - |
| Nathan 2002 | 40 | America | T:Oral midazolam 0.7 mg/kg (n=20) C:Oral midazolam 1.0 mg/kg (n=20) | - | - | T:2.50±0.03 C:2.50±0.03 | T:14±2 C:12±2 | Invasive | Cohort study | Success rate of sedation and hypnosis, time to fall asleep (min) |
| Schmalfuss 2005 | 326 | America | T:Oral midazolam mean dose 0.873 mg/kg (n=16) C:Oral chloral hydrate mean dose 65.2 mg/kg (n=310) | - | - | T:1.62±0.10 C:2.35±0.16 | T:12.3±4.9 C:12.5±4.8 | Noninvasive | Cohort study | Success rate of sedation and hypnosis, adverse reactions |
| Peretz 2014 | 23 | Israel | T:Oral midazolam 0.5 mg/kg (n=23) C:Oral midazolam 0.7 mg/kg (n=23) | Injection | 5 mg/1cc | - | - | Invasive | Cohort study | Degree of calm, time to fall asleep (min), adverse reactions |
| Aslıhan 2018 | 64 | - | T:Oral midazolam 0.5 mg/kg (n=32) C:Blank (n=31) | - | - | T:6.03±1.49 C:5.97±1.38 | T:21.88±5.37 C:22.59±7.02 | Invasive | Cohort study | Degree of calm |
| Gal Neuman 2018 | 1504 | Canada | T:Oral midazolam ＜0.3 mg/kg (n=33) C1:Oral midazolam 0.3 mg/kg-0.5 mg/kg (n=188) C2:Oral midazolam 0.5 mg/kg-0.7 mg/kg (n=1187) C3:Oral midazolam 0.7 mg/kg-0.9 mg/kg (n=59) C4:Blank (n=37) | - | - | T:3.5±2.4 C:4.2±2.7 | T:15.2±4.8 C:16.9±6.2 | - | Cohort study | Success rate of sedation and hypnosis |
| Sultan Keles 2018 | 52 | Turkey | T:Oral midazolam 0.5 mg/kg (n=28) C:Oral dexmedetomidine 2μg/kg (n=26) | - | - | T:5.1±1.4 C:5.3±2.3 | T:19.4±5.9 C:18.8±2.9 | Invasive | Cohort study | Success rate of sedation and hypnosis, success rate of mask acceptance, success rate of parental separation |
| D. Soy 1994 | 20 | Spain | T:Oral midazolam 0.3 mg/kg (n=20) | Oral solution | 1 mg/ml | 4-7 | - | Invasive | Case series | Success rate of sedation and hypnosis |
| Gianfranco Fraone 1999 | 61 | America | T:Oral midazolam 0.5 mg/kg (n=61) | - | - | 0.17-0.40 | - | Invasive | Case series | - |
| Nancy Kil 2003 | 24 | America | T:Oral midazolam 0.5 mg/kg (n=24) | - | - | 3.1 (1.6-5.4) | 16.06 (9.53-32.66) | Invasive | Case series | - |
| P.F. Day 2006 | 101 | UK and Australia | T1:Oral midazolam 0.25 mg/kg (n=5) T2:Oral midazolam 0.3 mg/kg (n=44) T3:Oral midazolam 0.5 mg/kg (n=33) T4:Oral midazolam 0.7 mg/kg (n=19) | - | - | UK 5.0±1.9 Australia 2.9±1.6 | - | Invasive | Case series | Success rate of sedation and hypnosis |
| Zeev N. Kain 2007 | 262 | America | T:Oral midazolam 0.5 mg/kg (n=262) | - | - | 5.68±2.46 | - | Invasive | Case series | Success rate of sedation and hypnosis |
| Jing quan 2009 | 10 | China | T:Oral midazolam 0.5 mg/kg (n=10) | - | - | 6.00±1.89 | 28.1±9.36 | Invasive | Case series | Time to fall asleep (min), adverse reactions |
| L. Lourenço-Matharu 2010 | 510 | UK | T:Oral midazolam 0.5 mg/kg (n=510) | - | - | 1.1-11.0 | ＜36 | Invasive | Case series | Adverse reactions |
| Jing quan 2010 | 109 | China | T:Oral midazolam 0.5 mg/kg-0.75 mg/kg (0．62±0．08) mg／kg (n=109) | Tablet | 15 mg/ tablet | 3.73±1.29 | 17.3±4.2 | Invasive | Case series | Success rate of sedation and hypnosis, adverse reactions |
| Xia bin 2010 | 23 | China | T:Oral midazolam 0.15 mg/kg-0.75 mg/kg (n=23) | - | - | 6.2 | - | Invasive | Case series | Time to fall asleep (min) |
| Sun lijun 2011 | 54 | China | T:Oral midazolam 0.5 mg/kg (n=54) | - | - | 6.2 (4-14) | - | Invasive | Case series | Adverse reactions |
| Ma lin 2012 | 30 | China | T:Oral midazolam 0.5 mg/kg-1.0 mg/kg (n=30) | - | - | 3.87±1.23 | 17.0±3.9 | Invasive | Case series | Success rate of sedation and hypnosis, adverse reactions |
| Jaya Dighe 2014 | 50 | India | T:Oral midazolam 0.5 mg/kg (n=50) | - | - | 1-10 | 13-23 | Invasive | Case series | Degree of calm, adverse reactions |
| Roberto B 2017 | 1 | Italy | T:Oral midazolam 0.5 mg/kg (n=1) | - | - | 7 | - | Invasive | Case report | Adverse reactions |

**Supplementary Table 2**

**Table 2:** Risk of bias in RCT studies

| **Study ID** | **Random sequence generation** | **Allocation concealment** | **Blinding of participants** | **Blinding of outcome assessment** | **Incomplete outcome data** | **Selective reporting** | **Other sources of bias** |
| --- | --- | --- | --- | --- | --- | --- | --- |
| Halim M Hennes 1990 | U | U | L | L | L | U | L |
| K.A.Payne 1991 | L | U | U | U | L | U | U |
| Mcmillan 1992 | U | U | U | L | L | U | U |
| Parnis 1992 | U | U | U | U | H | U | U |
| Weldon 1992 | U | U | U | U | H | H | U |
| Vetter 1993 | U | U | U | U | H | U | U |
| AIderson 1994 | L | U | L | U | L | U | L |
| Thomas S 1994 | L | U | H | U | L | L | L |
| Lyons1995 | U | U | H | L | L | U | U |
| Cray 1996 | L | U | L | L | L | U | U |
| Daniel A 1996 | L | U | L | L | L | U | U |
| Riva 1997 | U | U | U | L | L | U | U |
| V.Mitchell 1997 | U | U | U | L | L | U | U |
| Chris A. Liacouras 1998 | U | U | L | U | L | U | L |
| F C Davies 1998 | H | H | H | H | H | H | U |
| Dagostino 2000 | L | U | L | L | L | U | U |
| W. Funk 2000 | L | L | L | U | H | H | L |
| Luhmann 2001 | U | U | U | U | H | U | U |
| Wheeler 2001 | L | U | L | U | U | U | U |
| Younge 2001 | U | U | U | U | U | U | U |
| Charles J. Cote 2002 | U | U | L | U | L | U | L |
| Ian J.Everitt 2002 | U | U | H | U | L | U | L |
| Brosius KK 2002 | U | U | L | U | L | U | L |
| Wilson 2002 | U | H | H | H | U | U | U |
| Mcerlean 2003 | U | U | L | L | L | U | U |
| S.N.Wildt 2003 | L | U | U | L | L | U | U |
| Singh 2003 | U | U | L | U | H | U | H |
| Suranjit Debnath 2003 | U | U | U | U | L | U | L |
| Tamura 2003 | L | H | H | H | U | U | U |
| Brad Fine 2004 | U | U | L | L | U | H | L |
| Toshinori Horiuchi 2004 | U | U | H | L | H | U | L |
| Ilan Keidan 2005 | U | H | H | H | L | U | L |
| Mishra 2005 | U | U | U | L | L | U | U |
| Singh 2005 | L | U | L | L | L | U | L |
| Tamaporn P 2005 | U | U | U | L | L | U | U |
| Wan 2005 | U | U | H | U | L | U | L |
| Y-C.P.Aari 2005 | L | U | L | U | L | U | L |
| Bandana Koirala 2006 | U | U | U | U | L | U | L |
| K. E. Wilson 2006 | L | U | H | H | L | U | L |
| Liu 2006 | U | U | H | H | L | H | L |
| Selman Vefa Yildirim 2006 | L | U | L | H | L | U | L |
| Andre 2007 | L | U | L | L | U | U | U |
| Mccluskey 2007 | U | U | U | U | L | U | U |
| Simonetta Tesoro 2007 | H | U | U | U | H | U | L |
| Damle S.G 2008 | U | U | L | U | L | U | L |
| Chen 2009 | U | U | L | U | L | U | L |
| Mark D. Talon 2009 | L | U | H | U | H | U | L |
| Kajal Jain 2010 | L | L | L | L | L | U | L |
| Mandana Rafeey 2010 | U | U | H | H | H | U | L |
| Peter Templeton 2010 | L | L | L | L | L | U | L |
| Genoa G. FergUson 2011 | U | L | L | L | L | U | L |
| M. Ghali 2011 | L | L | L | L | L | U | U |
| Mehrdad Shoroghi 2011 | L | L | L | L | L | U | L |
| Mostafa Somri 2011 | U | L | U | U | L | U | L |
| MoUntain B W 2011 | U | U | L | L | L | U | U |
| Özcengiz D 2011 | L | U | U | L | L | U | U |
| Shabbir A 2011 | U | U | U | U | U | U | L |
| Hua 2012 | U | U | H | H | L | U | L |
| Chandni Sinha 2012 | L | U | L | U | L | U | L |
| Ashrafi 2013 | U | U | L | U | L | U | U |
| Hojjat Derakhshanfar 2013 | U | U | U | U | L | U | U |
| Isabelita D Uarte A 2013 | L | U | L | L | U | U | U |
| ThakUrta 2013 | L | U | U | U | U | U | U |
| Tyagi 2013 | U | U | L | L | U | U | U |
| Wang 2013 | U | L | L | U | L | U | L |
| Liu 2014 | L | U | L | H | L | U | L |
| Zhai 2014 | U | U | H | H | H | H | L |
| Abhishek K Phadke 2014 | L | U | H | H | L | U | U |
| Azarfar 2014 | U | U | U | U | L | U | U |
| B. Linares Segovia 2014 | L | U | L | U | L | U | U |
| Savla 2014 | L | L | L | L | L | L | L |
| SUjata ChaUdhary 2014 | L | U | U | U | L | L | U |
| SUman Arora 2014 | L | L | L | L | L | U | L |
| faritUs 2015 | U | U | U | U | L | U | U |
| Fatma BB 2015 | L | U | L | L | L | U | U |
| Hisham Y 2015 | L | U | U | L | L | U | U |
| Song 2016 | H | H | H | U | H | U | L |
| Ahmad Khodadad 2016 | U | U | L | U | U | L | U |
| Babita Ghai 2016 | L | U | L | L | L | U | U |
| Omar Hijazi 2016 | L | U | L | L | L | U | U |
| Orit RUbinstein 2016 | L | L | L | L | L | L | U |
| Forod Salehi 2017 | U | U | L | U | H | U | L |
| Li 2017 | U | U | L | U | L | U | L |
| M Kavya PrabhU 2017 | L | U | L | L | L | U | L |
| Sarika KUmari 2017 | L | U | L | L | U | U | L |
| KolathU 2018 | L | U | L | L | H | U | U |
| MadhUri S KUrdit 2018 | L | L | L | U | L | U | L |
| Reyhaneh Faghihian 2018 | L | U | L | H | L | U | L |
| You 2018 | U | U | H | H | L | H | L |

Note: H: high risk; L: low risk; U: unclear

**Supplementary Table 3**

**Table 3:** Risk of bias in Cohort studies

| **Study ID** | **Representativeness of the exposed cohort** | **Selection of the non exposed cohort** | **Ascertainment of exposure** | **Demonstration that outcome of interest was not present at start of study** | **Comparability of cohorts on the basis of the design or analysis** | **Assessment of outcome** | **Was follow-up long enough for outcomes to occur** | **Adequacy of follow up of cohorts** | **Sum** |
| --- | --- | --- | --- | --- | --- | --- | --- | --- | --- |
| Jack S 1995 | 1 | 1 | 0 | 0 | 1 | 0 | 1 | 1 | 5 |
| Jason J 2001 | 1 | 1 | 1 | 0 | 2 | 1 | 0 | 1 | 7 |
| Nathan 2002 | 0 | 1 | 0 | 1 | 0 | 0 | 1 | 1 | 4 |
| Schmalfuss 2005 | 1 | 1 | 1 | 0 | 2 | 0 | 1 | 1 | 7 |
| Peretz 2014 | 1 | 1 | 0 | 0 | 2 | 0 | 1 | 1 | 6 |
| Aslıhan 2018 | 0 | 1 | 1 | 1 | 1 | 0 | 0 | 0 | 4 |
| Gal 2018 | 1 | 1 | 1 | 0 | 0 | 1 | 0 | 1 | 5 |
| Sultan 2018 | 1 | 1 | 1 | 1 | 2 | 1 | 0 | 1 | 8 |
|  |  |  |  |  |  |  |  |  |  |

**Supplementary Table 4**

**Table 4:** Quality evaluation results of the case series

| **Study ID** | **Were there clear criteria for inclusion in the case series?** | **Was the condition measured in a standard, reliable way for all participants included in the case series?** | **Were valid methods used for identification of the condition for all participants included in the case series?** | **Did the case series have consecutive inclusion of participants?** | **Did the case series have complete inclusion of participants?** | **Was there clear reporting of the demographics of the participants in the study?** | **Was there clear reporting of clinical information of the participants?** | **Were there clear criteria for inclusion in the case series?** | **Was the condition measured in a standard, reliable way for all participants included in the case series?** | **Were valid methods used for identification of the condition for all participants included in the case series?** |
| --- | --- | --- | --- | --- | --- | --- | --- | --- | --- | --- |
| D.Soy 1994 | N | N | N | N | N | N | N | Y | N | Unclear |
| Gianfranco Fraone 1999 | Y | N | Not applicable | N | N | N | N | Y | N | Unclear |
| Nancy Kil 2003 | Y | N | Not applicable | N | N | N | N | N | N | Unclear |
| P.F.Day 2006 | Y | N | Not applicable | Y | Y | N | N | N | N | Unclear |
| Zeev N.Kain 2007 | Y | N | Not applicable | N | N | Y | N | N | N | Unclear |
| Jing Quan 2009 | Y | N | Not applicable | Y | Unclear | N | N | N | N | Unclear |
| Jing Quan 2010 | Y | N | Not applicable | Y | Unclear | N | N | N | N | Unclear |
| L.Lourenço-Matharu 2010 | Y | N | Not applicable | Y | Y | N | N | Y | N | Unclear |
| Xia Bin 2010 | N | N | Not applicable | N | N | N | N | N | N | Unclear |
| Sun Lijun 2011 | Y | N | Not applicable | Y | Y | N | N | N | N | Unclear |
| Ma Lin 2012 | Y | N | Not applicable | Y | Unclear | N | N | N | N | Unclear |
| Jaya Dighe 2014 | Y | N | N | N | N | N | N | Y | N | Unclear |

Note: Y: yes; N:no

**Supplementary Table 5**

**Table 5:** Quality evaluation results of the case reports

| **Study ID** | **Were patient’s demographic characteristics clearly described?** | **Was the patient’s history clearly described and presented as a timeline?** | **Was the current clinical condition of the patient on presentation clearly described?** | **Were diagnostic tests or assessment methods and the results clearly described?** | **Was the intervention(s) or treatment procedure(s) clearly described?** | **Was the post-intervention clinical condition clearly described?** | **Were adverse events (harms) or unanticipated events identified and described?** | **Does the case report provide takeaway lessons?** |
| --- | --- | --- | --- | --- | --- | --- | --- | --- |
| Roberto Bernardini 2017 | Y | Y | Y | Not applicable | Y | Y | Y | Y |

Note: Y: yes; N:no

**Supplementary Search strategy**

**Pubmed Search**

((((((Dormicum[Title/Abstract]) OR Versed[Title/Abstract]) OR Midazolam[Title/Abstract])) OR "Midazolam"[Mesh])) AND (((((((((("Child"[Mesh]) OR child[Title/Abstract])) OR ((("Infant, Newborn"[Mesh]) OR infant[Title/Abstract]) OR Newborn[Title/Abstract])) OR toddler[Title/Abstract]) OR (("Adolescent"[Mesh]) OR ((((((((((((((((Adolescents[Title/Abstract]) OR Adolescence[Title/Abstract]) OR Teens[Title/Abstract]) OR Teen[Title/Abstract]) OR Teenagers[Title/Abstract]) OR Teenager[Title/Abstract]) OR Youth[Title/Abstract]) OR Youths[Title/Abstract]) OR Adolescents, Female[Title/Abstract]) OR Adolescent, Female[Title/Abstract]) OR Female Adolescent[Title/Abstract]) OR Female Adolescents[Title/Abstract]) OR Adolescents, Male[Title/Abstract]) OR Adolescent, Male[Title/Abstract]) OR Male Adolescent[Title/Abstract]) OR Male Adolescents[Title/Abstract]))) OR (("Pediatrics"[Mesh]) OR ((pediatric[Title/Abstract]) OR pediatrics[Title/Abstract]))))))

**EMBASE search**

#17. #15 AND #16

#16. #1 OR #2 OR #3 OR #4

#15. #5 OR #6 OR #7 OR #8 OR #9 OR #10 OR #11 OR #12 OR #13 OR #14

#14. 'pediatric':ab,ti AND [embase]/lim

#13. 'adolescent':ab,ti AND [embase]/lim

#12. 'teenager':ab,ti AND [embase]/lim

#11. 'toddler':ab,ti AND [embase]/lim

#10. 'neonate':ab,ti AND [embase]/lim

#9. 'infant':ab,ti AND [embase]/lim

#8. 'newborn':ab,ti AND [embase]/lim

#7. 'children':ab,ti AND [embase]/lim

#6. 'child':ab,ti AND [embase]/lim

#5. 'child'/exp AND [embase]/lim

#4. 'versed':ab,ti AND [embase]/lim

#3. 'dormicum':ab,ti AND [embase]/lim

#2. 'midazolam':ab,ti AND [embase]/lim

#1. 'midazolam'/exp AND [embase]/lim

**The Cochrane Library search**

1 MeSH descriptor: [Midazolam] explode all trees

2 "midazolam":ti,ab,kw or Dormicum:ti,ab,kw or Versed:ti,ab,kw (Word variations have been searched)

3 MeSH descriptor: [Child] explode all trees

4 MeSH descriptor: [Infant, Newborn] explode all trees

5 MeSH descriptor: [Infant] explode all trees

6 MeSH descriptor: [Infant, Newborn] explode all trees

7 MeSH descriptor: [Adolescent] explode all trees

8 MeSH descriptor: [Adolescent] explode all trees

9 neonate:ti,ab,kw or toddler:ti,ab,kw or child:ti,ab,kw or newborn:ti,ab,kw or infant:ti,ab,kw (Word variations have been searched)

10 neonate:ti,ab,kw or toddler:ti,ab,kw or child:ti,ab,kw or newborn:ti,ab,kw or infant:ti,ab,kw (Word variations have been searched)

11 teenager:ti,ab,kw or adolescent:ti,ab,kw or pediatric:ti,ab,kw (Word variations have been searched)

12 #1 or #2

13 #3 or #4 or #5 or #6 or #7 or #8 or #9 or #10 or #11

14 #12 and #13

**CINAHL search**

(TI midazolam OR AB midazolam OR SU midazolam OR AB versed OR TI versed OR AB Dormicum OR TI Dormicum) AND (TI newborn OR TI neonate OR TI infant OR TI child OR TI toddler OR TI teenager OR TI adolescent OR TI pediatrics OR AB newborn OR AB neonate OR AB infant OR AB child OR AB toddler OR AB teenager OR AB adolescent OR AB pediatrics)

**International Pharmaceuticals search**

1 Midazolam.mp. [mp=title, subject heading word, registry word, abstract, trade name/generic name]

2 Dormicum.mp. [mp=title, subject heading word, registry word, abstract, trade name/generic name]

3 Versed.mp. [mp=title, subject heading word, registry word, abstract, trade name/generic name]

4 1 or 2 or 3

5 child.mp. [mp=title, subject heading word, registry word, abstract, trade name/generic name]

6 newborn.mp. [mp=title, subject heading word, registry word, abstract, trade name/generic name]

7 infant.mp. [mp=title, subject heading word, registry word, abstract, trade name/generic name]

8 neonate.mp. [mp=title, subject heading word, registry word, abstract, trade name/generic name]

9 toddler.mp. [mp=title, subject heading word, registry word, abstract, trade name/generic name]

10 teenager.mp. [mp=title, subject heading word, registry word, abstract, trade name/generic name]

11 adolescent.mp. [mp=title, subject heading word, registry word, abstract, trade name/generic name]

12 pediatric.mp. [mp=title, subject heading word, registry word, abstract, trade name/generic name]

13 5 or 6 or 7 or 8 or 9 or 10 or 11 or 12

14 4 and 13

**CNKI search**

SU=('咪达唑仑'+'咪唑安定'+'速眠安'+'力月西'+'瑞太'+'多美康')*('儿童'+'新生儿'+'婴儿'+'幼儿'+'儿科'+'青少年')

**VIP search**

（M=咪唑安定 OR M=咪达唑仑 OR M=速眠安OR M=力月西 OR M=瑞太OR M=多美康） AND (M=儿童 OR M=新生儿OR M=婴儿 OR M=幼儿 OR M=儿科 OR M=青少年)

**Wanfang search**

(主题:(咪达唑仑)+主题:(咪唑安定)+主题:(速眠安)+主题:(力月西)+主题:(瑞太)+主题:(多美康))*(主题:(儿童)+主题:(新生儿)+主题:(婴儿)+主题:(幼儿)+主题:(儿科) +主题:(青少年))

**CBM search**

19 #18 and #7 -限定:-

18 #17 or #16 or #15 or #14 or #13 or #12 or #11 or #10 or #9 or #8 -限定:-

17 主题词:儿科学/全部树/全部副主题词 -限定:-

16 主题词:青少年/全部树/全部副主题词 -限定:-

15 主题词:婴儿, 新生/全部树/全部副主题词 -限定:-

14 主题词:儿童/全部树/全部副主题词 -限定:-

13 缺省[智能]:儿科 -限定:-

12 缺省[智能]:青少年 -限定:-

11 缺省[智能]:幼儿 -限定:-

10 缺省[智能]:婴儿 -限定:-

9 缺省[智能]:新生儿 -限定:-

8 缺省[智能]:儿童 -限定:-

7 #6 or #5 or #4 or #3 or #2 or #1 -限定:-

6 缺省[智能]:咪唑安定 -限定:-

5 主题词:咪达唑仑/全部树/全部副主题词 -限定:-

4 缺省[智能]:力月西 -限定:-

3 缺省[智能]:咪达唑仑 -限定:-

2 缺省[智能]:多美康 -限定:-

1 缺省[智能]:瑞太 -限定:-

**Reference**

Arai, Y. C., Fukunaga, K., and Hirota, S. (2005). Comparison of a combination of midazolam and diazepam and midazolam alone as oral premedication on preanesthetic and emergence condition in children. *Acta Anaesthesiol. Scand*. 49, 698–701. doi: 10.1111/j.1399-6576.2005.00700.x

Belen, F. B., Kocak, U., Kayilioglu, H., Isik, M., Keskin, E. Y., Oner, N., et al. (2015). Use of low dose oral midazolamduring invasive procedures in pediatric hematology patients. *Gazi. Med. J.* 26, 177–179. doi: 10.12996/gmj.2015.52

Brosius, K. K., and Bannister, C. F. (2002). Oral midazolam premedication in preadolescents and adolescents. *Anesth. Analg*. 94, 31–36. doi: 10.1213/00000539-200201000-00006

Coté, C. J., Cohen, I. T., Suresh, S., Rabb,M., Rose, J. B.,Weldon, B. C., et al. (2002). A comparison of three doses of a commercially prepared oral midazolam syrup in children. *Anesth. Analg*. 94, 37–43. doi: 10.1213/00000539-200201000-00007

Cray, S. H., Dixon, J. L., Heard, C. M. B., and Selsby, D. S. (1996). Oral midazolam premedication for paediatric day case patients. *Pediatr. Anesth*. 6, 265–270.

Damle, S. G., Gandhi, M., and Laheri, V. (2008). Comparison of oral ketamine and oral midazolam as sedative agents in pediatric dentistry. *J. Indian. Soc. Pedod. Prev. Dent*. 26, 97–101. doi: 10.4103/0970-4388.43186

Davies, F. C., and Waters, M. (1998). Oral midazolam for conscious sedation of children during minor procedures. *J. Accid. Emerg. Med*. 15, 244–248. doi: 10.1136/emj.15.4.244

de Wildt, S. N., Kearns, G. L., Sie, S. D., Hop, W. C., and van den Anker, J. N. (2003). Pharmacodynamics of intravenous and oral midazolam in preterm infants. *Clin. Drug Invest*. 23, 27–38. doi: 10.2165/00044011-200323010-00004

Everitt, I. J., and Barnett, P. (2002). Comparison of two benzodiazepines used for sedation of children undergoing suturing of a laceration in an emergency department. *Pediatr. Emerg. Care* 18, 72–74. doi: 10.1097/00006565-200204000-00002

Faghihian, R., Eshghi, A., Faghihian, H., and Kaviani, N. (2018). Comparison of oral melatonin and midazolam as premedication in children undergoing general anesthesia for dental treatment. *Anesth. Pain. Med*. 8:e64236. doi: 10.5812/aapm.64236

Ferguson, G. G., Chen, C., Yan, Y., Royer, M. E., Campigotto, M., Traxel, E. J., et al. (2011). The efficacy of oral midazolam for decreasing anxiety in children undergoing voiding cystourethrogram: a randomized, double-blind, placebo controlled study*. J. Urol.* 185, 2542–2546. doi: 10.1016/j.juro.2011.01.031

Fine, B., Castillo, R., McDonald, T., Paisansathan, C., Zsigmond, E., and Hoffman, W. E. (2004). Jet injector compared with oral midazolam for preoperative sedation in children. *Pediatr. Anesth.* 14, 739–743. doi: 10.1111/j.1460-9592.2004.01294.x

Hennes, H. M., Wagner, V., Bonadio, W. A., Glaeser, P. W., Losek, J. D., Walsh-Kelly, C. M., et al. (1990). The effect of oral midazolam on anxiety of preschool children during laceration repair. *Ann. Emerg. Med*. 19, 1006–1009.

Horgesheimer, J. J., Pribble, C. G., and Lugo, R. A. (2001). The effect of midazolam premedication on discharge time in pediatric patients undergoing general anesthesia for dental restorations. *Pediatr. Dent*. 23, 491–494.

Koirala, D. B., Pandey, P. R., Saksen, P. A., Kumar, D. R., and Sharma, D. S. (2006). A comparative evaluation of newer sedatives in conscious sedation. *J. Clin. Pediatr. Dent*. 30, 273–276. doi: 10.17796/jcpd.30.4.540025283p827511

Kurdi, M. S., and Muthukalai, S. P. (2016). A comparison of the effect of two doses of oral melatonin with oral midazolam and placebo on pre-operative anxiety, cognition and psychomotor function in children: a randomised double-blind study. *Indian. J. Anaesth*. 60, 744–750. doi: 10.4103/0019-5049.191688

Liu, J. (2006).Observation on the effect of midazolamin children before anesthesia. *Pract. Clin. Med*. 7, 118–118. doi: 10.3969/j.issn.1009-8194.2006.02.048

Neuman, G., Tobia, R. S., Koren, L., Leiba, R., and Shavit, I. (2018). Single dose oral midazolam for minor emergency department procedures in children: a retrospective cohort study. *J. Pain Res*. 11, 319–324. doi: 10.2147/JPR.S156080

Özcengiz, D., Gunes, Y., and Ozmete, O. (2011). Oral melatonin, dexmedetomidine, and midazolam for prevention of postoperative agitation in children*. J. Anesth*. 25, 184–188. doi: 10.1007/s00540-011-1099-2

Parnis, S. J., Foate, J. A., van der Walt, J. H., Short, T., and Crowe, C. E. (1992). Oral midazolam is an effective premedication for children having day-stay anaesthesia. *Anaesth. Intens. Care* 20, 9–14. doi: 10.1177/0310057X9202000102

Prabhu, M. K., and Mehandale, S. G. (2017). Comparison of oral dexmedetomidine versus oral midazolam as premedication to prevent emergence agitation after sevoflurane anaesthesia in paediatric patients. *Indian J. Anaesth.* 61, 131–136. doi: 10.4103/0019-5049.199852

Rafeey, M., Ghojazadeh, M., Feizo Allah Zadeh, H., and Majidi, H. (2010). Use of oral midazolam in pediatric upper gastrointestinal endoscopy. *Pediatr. Int*. 52, 191–195. doi: 10.1111/j.1442-200X.2009.02936.x

Riva, J., Lejbusiewicz, G., Papa, M., Lauber, C., Kohn, W., Da Fonte, M., et al. (1997). Oral premedication with midazolam in paediatric anaesthesia. Effects on sedation and gastric contents. *Pediatr. Anesth*. 7, 191–196. doi: 10.1046/j.1460-9592.1997.d01-75.x

Savla, J. R., Ghai, B., Bansal, D., and Wig, J. (2014). Effect of intranasal dexmedetomidine or oral midazolam premedication on sevoflurane EC 50 for successful laryngeal mask airway placement in children: a randomized, double-blind, placebo-controlled trial. *Pediatr. Anesth.* 24, 433–439. doi: 10.1111/pan.12358

Segovia, B. L., Cuevas, M. G., Casillas, I. R., Romero, J. G., Buenrostro, I. B., Torres, R. M., et al. (2014). Pre-anaesthetic medication with intranasal dexmedetomidine and oral midazolamas an anxiolytic. A clinical trial. *An. Esp. Pediatr*. 81, 226–231. doi: 10.1016/j.anpede.2013.12.003

Shabbir, A., Bhat, S., Sundeep, H., and Salman, S. M. (2011). Comparison of oral midazolam and triclofos in conscious sedation of uncooperative children. *J. Clin. Pedatr. Dent*. 36, 189–196. doi: 10.17796/jcpd.36.2.0346178414pvw865

Shoroghi, M., Arbabi, S., Farahbakhsh, F., Sheikhvatan, M., and Abbasi, A. (2011). Perioperative effects of oral midazolam premedication in children undergoing skin laser treatment: a double-blinded randomized placebo-controlled trial. *Acta. Cir. Bras*. 26, 303–309. doi: 10.1590/S0102-86502011000400010

Song, Y. A., Gu, H. B., Zhang, J.W., Huan, Y., and Zhang, M. Z. (2016). The effect analysis of oral midazolam decreasing dissociative anxiety in surgical children. *Prog. Mod. Biomed*. 16, 3942–3944. doi: 10.13241/j.cnki.pmb.2016.20.039

Tamura, M., Nakamura, K., Kitamura, R., Kitagawa, S., Mori, N., and Ueda, Y. (2003). Oral premedication with fentanyl may be a safe and effective alternative to oral midazolam. *Eur. J. Anaesthesiol.* 20, 482–486. doi: 10.1097/00003643-200306000-00011

Templeton, P., Burton, D., Cullen, E., Lewis, H., Allgar, V., and Wilson, R. (2010). Oral midazolam for removal of Kirschner wires in the children’s orthopaedic outpatient department: a randomized controlled trial. *J. Pediatr. Orthoped*. 30, 130–134. doi: 10.1097/BPO.0b013e3181ced3ae

Tesoro, S., Vicchio, N., Marchesini, L., and Aldo Peduto, V. (2007). Sedation of children with ADHD: trazodone or midazolam? *Pediatr. Anesth*. 17, 1008–1009. doi: 10.1111/j.1460-9592.2007.02267.x

Tyagi, P., Tyagi, S., and Jain, A. (2013). Sedative effects of oral midazolam, intravenous midazolam and oral diazepam in the dental treatment of children. *J. Dent. Child*. 37, 301–306. doi: 10.17796/jcpd.37.3.6u482603r0388558

Vetter, T. R. (1993). A comparison of midazolam, diazepam, and placebo as oral anesthetic premedicants in younger children. *J. Clin. Anesth*. 5, 58–61. doi: 10.1016/0952-8180(93)90090-2

Wang, W., Yan, M., Lei, L.P., Yu, L. N., and Zhang, F. J. (2013). Efficacy of midazolam premedication for prevention of emergence agitation after sevoflurane anesthesia in children undergoing minor surgery. *Chin. J. Anesthesiol*. 33, 541–543.

Wilson, K. E., Girdler, N. M., and Welbury, R. R. (2006). A comparison of oral midazolam and nitrous oxide sedation for dental extractions in children. *Anaesthesia* 61, 1138–1144. doi: 10.1111/j.1365-2044.2006.04835.x

Wilson, K. E., Welbury, R. R., and Girdler, N. M. (2002). A study of the effectiveness of oral midazolam sedation for orthodontic extraction of permanent teeth in children: a prospective, randomised, controlled, crossover trial*. Br. Dent. J*. 192, 457–462. doi: 10.1038/sj.bdj.4801400
